# Enhancing Privacy in IoT-Enabled Digital Infrastructure: Evaluating Federated Learning for Intrusion and Fraud Detection

**DOI:** 10.3390/s25103043

**Published:** 2025-05-12

**Authors:** Amogh Deshmukh, Peplluis Esteva de la Rosa, Raul Villamarin Rodriguez, Sandeep Dasari

**Affiliations:** 1 School of Technology, Woxsen University, Hyderabad 502345, India; peplluis.esteva@woxsen.edu.in (P.E.d.l.R.); dasari.sandeep@woxsen.edu.in (S.D.); 2 School of Business, Woxsen University, Hyderabad 502345, India; raul.rodriguez@woxsen.edu.in

**Keywords:** artificial intelligence, cyber security, deep learning, federated learning, intrusion detection

## Abstract

Challenges in implementing machine learning (ML) include expanding data resources within the finance sector. Banking data with significant financial implications are highly confidential. Diverse breaches and privacy violations can result from a combination of user information from different institutions for banking purposes. To address these issues, federated learning (FL) using a flower framework is utilized to protect the privacy of individual organizations while still collaborating through separate models to create a unified global model. However, joint training on datasets with diverse distributions can lead to suboptimal learning and additional privacy concerns. To mitigate this, established FL algorithms such as federated averaging (FedAvg), federated proximal (FedProx), and federated optimization (FedOpt), previously introduced in the literature, are evaluated in our study. These methods work with data locality during training at local clients without exposing data, while maintaining global convergence to enhance the privacy of local models within the framework. In this analysis, the UNSW-NB15 and credit datasets were employed, utilizing precision, recall, accuracy, F1-score, ROC, and AUC as performance indicators to demonstrate the effectiveness of applying FedAvg, FedProx, and FedOpt within the flower framework. The algorithms considered were subjected to an empirical study, which revealed significant performance benefits when using the flower framework. Consequently experiments were conducted over 50 rounds using the UNSW-NB15 dataset, which achieved accuracies of 99.87% for both FedAvg and FedProx and 99.94% for FedOpt. Similarly, with the credit dataset under the same conditions, FedAvg and FedProx achieved accuracies of 99.95% and 99.94%, respectively, while FedOpt also achieved comparable accuracy. These simulation-based results suggest that the proposed framework is effective on widely used benchmark datasets and, with further validation in deployed environments, has the potential to support secure and privacy-preserving collaborative machine learning across various domains.

## 1. Introduction

In recent years, significant attention has been placed on the use of artificial intelligence, including ML and deep learning approaches, to solve real-world problems. The COVID-19 pandemic has highlighted the importance of strong digital connectivity, which has accelerated the global use of digital financial assets and electronic transactions. As economies grow and financial data volumes increase, financial institutions use ML for analytics to make informed decisions and enhance growth. AI improves operations across sectors such as finance. This study examines how analyzing financial data helps organizations make informed decisions. However, the sharing of information between entities raises significant data privacy concerns. Collaborative learning addresses this issue by allowing multiple agents to compute their data and contribute to a model without revealing underlying data. FL is an effective approach that helps clients to participate in an ML process using local data. Clients train models locally and share updates that are aggregated to enhance a global model and preserve data privacy while leveraging diverse distributed datasets. FL is advantageous in environments with heterogeneous data, devices, settings, and communication protocols, a property that has been highlighted in the foundational FL literature [1]. In today’s interconnected business ecosystems, collaboration among organizations, such as supply chains, is common. FL accommodates the inherent heterogeneity in such collaborations, allowing organizations to derive collective intelligence while keeping data confidential. This enables participants to gain insights and make informed decisions without compromising their privacy.

Among the benefits that FL offers are data privacy, scalability, resource optimization, and collaborative intelligence. Privacy risk is reduced, and clients maintain their data locally. By leveraging cloud computing and distributed systems, the scalability and utilization of resources can be boosted by the contribution of multiple clients. Shared knowledge is beneficial for organizations that do not expose sensitive information. However, federated learning has several challenges. The performance is affected by resource constraints that arise from differences in computational capabilities across participating devices. Balancing model utility with data privacy can present a challenging privacy–utility trade-off. Adaptive processing approaches are needed to address data heterogeneity, and computational complexity may have an impact on performance. However, FL is a versatile solution to problems in banking, insurance, healthcare, and government. FL must also address privacy concerns, data heterogeneity, and various limitations. To address these challenges, we used algorithms such as FedAvg, FedProx, and FedOpt, which facilitate collaborative data analysis while preserving privacy. In this study, we investigated a flower framework. Flower is designed to be flexible and resilient to support FL tasks across heterogeneous applications, datasets, and models. This allows businesses to learn from each other without compromising data privacy. It comes with reusable components and workflows that apply to any domain, and they are scalable, provide data integrity, and secure client data privacy via standardized security measures. A flower framework is an optimal solution for efficient and scalable collaborative learning by managing several data modalities and heterogeneities.

The flower framework is built upon the principles that make collaborative learning practical. Ease of use is emphasized through access to pre-built FL components for streamline operations. It is flexible: it works with various types of data, contexts, and devices. This includes mechanisms for anonymizing clients with confidentiality and privacy. Communication protocol enhancement between participants is more efficient, and handling heterogeneity guarantees robust and reliable learning processes. These principles ensure that data privacy remains intact, allowing organizations to collaborate without client data leaving their premises or compromising sensitive information.

This study focuses on utilizing the flower framework to evaluate various FL algorithms in solving problems such as intrusion detection [2] and fraud detection [3] in credit cards. The key contributions of this study are as follows:

1. Methodology Proposal: Introducing a methodology based on the flower framework for empirical studies of existing FL algorithms using diverse datasets.
2. Algorithm Integration: Integrating well-established FL algorithms (FedAvg, FedProx, and FedOpt, as originally introduced in the FL literature) into the flower framework together with our enhanced CNN model (IIDNet), and configuring them for the non-IID data encountered in intrusion and fraud detection. We do not claim novelty of the underlying algorithms themselves; the novelty lies in their joint implementation and empirical study within this framework.
3. Empirical Evaluation: Conducting an empirical study with a prototype application, executing different algorithms within the framework, and evaluating their performance.

The remainder of the paper is organized as follows: Section 2 provides related work on collaborative learning, including standard and deep learning methods. Section 3 presents the preliminaries necessary for understanding the proposed methodology and the adaptation of the flower framework. Section 4 details the proposed method and the underlying algorithms for evaluating FL efficiency within the flower framework. Section 5 discusses the experimental setup, results, and performance comparisons. Finally, Section 6 concludes the paper and outlines potential future work in collaborative learning.

## 2. Related Works

FL has gained recognition as a promising approach for enhancing privacy and security in machine learning applications, particularly in intrusion detection and fraud detection systems. In this section, we review relevant studies in this area, highlighting how each work differs from our research and explain the unique contributions of our study in comparison to existing literature.

Intrusion detection systems (IDS) and financial fraud detection are critical areas in cybersecurity [4] and finance, respectively. Traditional solutions rely on centralized machine learning models [5], which pose several challenges, such as data privacy concerns, scalability issues, and communication overhead. FL has evolved as a viable solution to overcome these challenges by allowing distributed model training without data sharing. Early and classical approaches to IDS were based on signature or anomaly-based detection methods. However, these centralized approaches face several challenges, as follows:

- Data Centralization and Privacy Risks: Traditional IDS architectures require aggregating network traffic data into a central location, exposing sensitive information and creating attractive targets for adversaries.
- Scalability Issues: As network sizes grow, the centralized processing of vast volumes of data leads to increased latency and reduced responsiveness.
- False Positives/Negatives: The accuracy of anomaly detection methods often suffers due to imbalanced data distributions and a lack of contextual information from diverse environments. These constraints emphasize the necessity for a more secure and distributed approach to IDS that does not compromise on detection accuracy.

Recent advances in FL have demonstrated potential in overcoming the challenges. Identified in both IDS and fraud detection domains. FL enables multiple institutions to train a model collaboratively without exposing raw data. Key contributions in this area include the following:

- FedAvg: A foundational algorithm that aggregates locally trained models to achieve a global model without data sharing.
- FedProx: An extension of FedAvg designed to handle heterogeneity among clients, which is particularly important when dealing with diverse financial and network environments.
- Federated Optimization Methods: These approaches focus on ensuring convergence and stability even when local data distributions vary significantly.

The literature consistently highlights that FL can reduce privacy risks and improve scalability, but it also points out that heterogeneous data distributions can lead to suboptimal convergence if not carefully managed.

### 2.1. Summary and Relevance to the Current Study

The reviewed literature demonstrates that while significant progress has been made in applying machine learning to IDS and fraud detection, key limitations persist, especially regarding data privacy, scalability, and model generalization. Our work builds on this foundation in the following ways:

- Integrating FL with Domain-Specific Requirements: The study leverages the flower framework to implement FL strategies that protect data privacy while enabling collaborative learning across multiple institutions.
- Addressing Data Heterogeneity: By comparing methods such as FedAvg, FedProx, and FedOpt, our study explicitly tackles the issue of non-IID data distributions, a common challenge in both financial fraud detection and IDS.
- Demonstrating Real-World Viability: Through empirical studies using the UNSW-NB15 [6] and credit datasets [7], the study validates that our approach not only overcomes the traditional limitations but also achieves high performance in practical scenarios.

### 2.2. Federated Learning Methods for Intrusion and Fraud Detection

Several researchers have explored the application of FL in IDS and fraud detection [8] by employing various architectures [9] and algorithms.

Sun et al. [10] proposed a hierarchical FL-based IDS for 5G smart grids, utilizing transformer-based models to enhance detection performance. Their work focused on a specific domain—smart grids—and leverages a hierarchical FL architecture to address the unique challenges in that context. In contrast, our study employs the flower framework to evaluate standard FL algorithms (FedAvg, FedProx, and FedOpt) in a broader context, addressing both intrusion and fraud detection across diverse datasets. Hamdi [11] investigated an FL-based IDS for the Internet of Things (IoT), emphasizing per-client evaluation to understand privacy-performance trade-offs. While this work provides insights into individual client contributions in FL, it does not focus on comparing multiple FL algorithms or using a specific framework such as flower. Our research differs by providing a comparative analysis of several FL algorithms within the flower framework, assessing their performance in intrusion and fraud-detection tasks.

Begum et al. [12] introduced a blockchain-driven FL approach for intrusion detection in Internet of Medical Things (IoMT) networks. By integrating blockchain technology, they enhanced the security and transparency of the FL process. Although blockchain integration offers additional security benefits, our study focuses on evaluating FL algorithms without the complexity of blockchain, allowing us to specifically analyze the algorithm’s effectiveness within the flower framework. Yang et al. [13] proposed a federated learning method for credit card fraud detection that addresses data insufficiency issues by collaboratively training models across multiple entities. Their work demonstrated the potential of FL to improve fraud detection systems. Our study also tackles credit card fraud detection but differs by systematically evaluating FL algorithms using the flower framework and comparing their performance, thereby providing broader insights into the applicability of FL in fraud detection.

Kumar et al. [14] presented a privacy-preserving federated machine learning mechanism utilizing an artificial immune system-based IDS in edge computing environments. Although their research enhances data privacy and security using specialized techniques, our work focuses on standard FL algorithms and evaluates their performance within the framework, making our findings more generalizable to various domains. Lazzarini et al. [15] evaluated federated learning for IoT intrusion detection by comparing different aggregation algorithms, including FedAvg, FedAvgM, FedAdam, and FedAdagrad, using a shallow ANN model on the ToN_IoT and CICIDS2017 datasets in both binary and multiclass classification tasks. Their study focused on assessing the performance of these algorithms in a federated setting for intrusion detection. Similarly, Jithish et al. [16] proposed a federated learning-based approach for distributed anomaly detection in smart grids by implementing various machine learning models on resource-constrained edge devices. They compared the performance of federated models with centralized models and evaluated resource utilization, thereby demonstrating the feasibility of deploying federated learning in smart grid environments.

### 2.3. Use of the Flower Framework in Recent Studies

Recently, the flower framework has gained attention for its flexibility in implementing FL. Our study differs from previous works in that it offers a systematic comparison of multiple FL algorithms within a single, flexible framework. Unlike the study by Alazab et al. [17], which focused solely on FedAvg, we explored additional algorithms such as FedProx and FedOpt. Ruzafa-Alcázar et al. [18] investigated privacy-preserving techniques within the FL for industrial IoT applications. By utilizing different datasets associated with IoT environments, they demonstrated that the system could effectively discern intrusions while preserving data privacy. Their study emphasized the importance of confidentiality in collaborative learning but did not evaluate the performance of various FL algorithms or the use of specific frameworks such as flower.

Dasari and Kaluri [19] proposed a privacy-preserving approach in the financial sector using the flower framework, emphasizing the importance of data protection in collaborative learning. However, their work focused on privacy mechanisms without a comparative analysis of different FL algorithms. Our study fills this gap by assessing how various FL algorithms perform within the flower framework across different applications, thereby providing broader insights into the applicability and effectiveness of FL in security-sensitive domains. Chen et al. [20] explored communication-efficient federated deep learning with layer-wise asynchronous model updates, and implemented their approach using the flower framework. Their study focuses on optimizing communication efficiency in FL but did not specifically address intrusion or fraud detection.

Our research distinguishes itself by employing the flower framework to conduct an empirical evaluation of multiple FL algorithms—FedAvg, FedProx, and FedOpt—in the context of both intrusion and fraud detection. We utilized benchmark datasets (UNSW-NB15 for intrusion detection and a credit card dataset for fraud detection) to assess the performance of the algorithms, providing practical insights into the benefits and limitations of each algorithm when implemented within the flower framework.

### 2.4. Comparison with Existing Works

Table 1, provides a comparative analysis of related federated learning studies, highlighting the differences in FL architectures, algorithms employed, datasets used, and their key contributions. Li et al. [21] investigated the privacy aspects of FL, along with other parameters, such as model complexity, processing speed, and global accuracy across different clients. Their study also considered the costs involved and model convergence within the learning process using financial data for fraud detection as a case study. However, their work focused on a specific FL system without comparing multiple FL algorithms or utilizing a framework that can be generalized across different applications.

In contrast to these studies, our research provides a comprehensive empirical analysis of several well-known FL algorithms—FedAvg, FedProx, and FedOpt—implemented within the flower framework. Specifically, it will examine how these algorithms perform in the context of intrusion detection and credit card fraud detection using the benchmark datasets UNSW-NB15 and a credit card transaction dataset. By evaluating the algorithms both with and without the flower framework, we demonstrate the framework’s effectiveness in enhancing the performance of FL algorithms across diverse datasets.

By conducting empirical studies on both intrusion detection and fraud detection tasks, we highlight the versatility of the flower framework in handling heterogeneous data and models. Our findings suggest that the flower framework enhances algorithm performance and facilitates scalability and privacy preservation, which are critical factors in the practical implementation of FL. This comprehensive evaluation contributes to a deeper understanding of how standard FL algorithms can be effectively employed within a flexible framework to improve security measures in digital infrastructure.

### 2.5. Deep Learning Based Methods

Deep learning models have been increasingly adopted in FL frameworks to enhance IDS and fraud detection mechanisms. In this subsection, we compare the deep learning models used in previous studies with the enhanced convolutional neural network (CNN) model employed in our research, highlighting the differences and unique contributions of our work. Li et al. [22] proposed DeepFed, a hybrid model combining a CNN and gated recurrent units (GRU) for cyber threat detection in industrial cyber–physical systems (CPS). They utilized a real-world gas pipeline system dataset and demonstrated that their model outperformed state-of-the-art methods in terms of accuracy, precision, recall, and F1-score. While their work focused on a specific industrial application and employed a hybrid CNN-GRU architecture, our study introduced an enhanced CNN model, known as IIDNet (intelligent intrusion detection network), designed to work effectively in both intrusion detection and fraud detection tasks across diverse datasets. Unlike the hybrid approach, our model leverages advanced CNN architectures to capture the spatial features relevant to different types of network traffic and transaction data.

Aouedi et al. [23] explored federated semi-supervised learning for attack detection in Industrial Internet of Things (IIoT) environments, using autoencoders (AE) and GRU models on a gas pipeline SCADA system dataset. Their approach improved the accuracy of attack classification compared to non-federated methods. However, their model primarily focused on anomaly detection using AEs and recurrent architectures. In contrast, our work employs an enhanced CNN model that directly addresses classification tasks in both intrusion and fraud detection and operates within the flower framework to leverage standard FL algorithms, thus enhancing scalability and applicability across different domains. Zhang et al. [24] developed a lightweight IDS for distributed IIoT environments by using ensemble classifiers and instance-based transfer learning within an FL framework. They evaluated their approach using the CIC-IDS2017 and CIC-IDS2018 datasets. Although their method emphasizes ensemble models and transfer learning techniques, our study introduces a deep learning model, IIDNet, which is specifically designed to capture intricate patterns in data using convolutional layers.

The intrusion detection and credit card fraud detection have been tested on our model in the flower framework, and Diro and Chilamkurti [25] used autoencoders and DBN on NSL-KDD to detect distributed attacks in IoT networks. Alkahtani and Aldhyani [26] used CNN, LSTM, and CNN-LSTM on IoTID20 to detect intrusions in the IoT, because Mothukuri et al. [27] used GRU and RF on a Modbus-based network to detect federated anomalies in the IoT. Popoola et al. [28] used DNN on Bot-IoT and N-BaIoT to detect zero-day botnet attacks in IoT-edge devices based on federated learning; therefore, the results are notable.

Our study’s unique contributions lie in the development of IIDNet, its implementation within the flower framework using standard FL algorithms, and its evaluation across diverse datasets, all of which advance the field of privacy-preserving collaborative machine learning in intrusion and fraud detection.

### 2.6. Advances in FL and ML Techniques for IDS

Another study on critical infrastructure IDS highlights that FL with vertically partitioned data achieves high accuracy but faces computational overhead owing to homomorphic encryption, underscoring the need for more efficient privacy-preserving techniques [29]. In IoT environments, convolutional neural networks have proven effective in detecting anomalies, achieving up to 99.51% and 92.85% accuracy on NID and BoT-IoT datasets, respectively [30]. The MULTI-BLOCK framework integrates machine learning with software-defined networking to enhance IoT security by synchronizing communication, traffic monitoring, and attack mitigation [31]. Addressing the need for modern datasets, the UNSW-NB15 dataset offers a comprehensive resource by combining real network activities with synthesized attacks and filling gaps left by older datasets such as KDD98 [32].

FL combined with transfer learning has been shown to improve IDS performance beyond 5G networks, achieving 99.57% accuracy on the CICIDS2017 dataset [33]. FL is advantageous over traditional approaches in handling non-IID data and managing communication overhead, although challenges such as security vulnerabilities persist [34]. Blockchain integration with FL ensures secure and transparent anomaly detection with minimal performance impact, particularly on datasets like CICIDS2017 [35]. Intrusion detection frameworks combining FL with transfer learning address resource constraints and achieve high accuracy while preserving privacy [36]. IoHT networks benefit from deep neural networks integrated with FL, ensuring data confidentiality with detection rates of 91.40% and 98.47% [37].

LSTM-based FL frameworks for Fog-IoT networks exhibit improved real-time mitigation of cyberattacks, as demonstrated on the BoT-IoT dataset [38]. The DPFL-F2IDS framework, with differential privacy and Adabound optimization, achieves high F1-scores for binary and multi-class classifications in vehicle networks [39]. Knowledge distillation techniques applied in semi-supervised FL frameworks for IoT intrusion detection improve performance, with CNN-based voting mechanisms enhancing the accuracy [40]. Surveys on FL for resource-constrained IoT devices address the challenges of limited computational power, communication overhead, and energy efficiency, thereby offering potential solutions [41]. Evaluations of NIDS models reveal differences between synthetic datasets, such as CIC-IDS2017, and real-world datasets, raising concerns about model generalizability [42]. FL frameworks in ambient assisted living environments enhance anomaly detection and privacy, achieving performance comparable to centralized deep learning models [43].

ML-based IDS for IoT networks using the UNSW-NB15 dataset with PCA for feature reduction demonstrated strong performance, with XGBoost achieving 99.99% accuracy [44]. Advanced IDS frameworks for IoT environments employ optimization algorithms such as spider monkey optimization with stacked-deep polynomial networks to effectively classify attacks such as DoS, U2R, and R2L [45]. This paper proposes an FL-based IDS for IoT networks that aim to preserve data privacy and provides high detection accuracy. Evaluated using the NSL-KDD dataset, the federated model achieved comparable results to centralized approaches while reducing communication overhead and addressing data privacy concerns [46]. The FedAdp algorithm accelerates convergence by assigning adaptive weights and enhancing training efficiency with non-IID data [47]. Explainable IDS frameworks using SHAP ensure interpretability and achieve high accuracy on the ToN_IoT dataset in IoV networks [48]. Finally, FedBatch, a CNN-MLP-based FL model for maritime transportation systems, addressed communication instability and achieved stable convergence and high accuracy on the NSL-KDD dataset [49].

The literature shows that FL is widely used in various application domains for collaborative learning and deriving actionable knowledge from the data of multiple sites or businesses in a particular domain. Different ML and deep learning techniques were applied in FL, and research has aimed to achieve security and privacy while benefiting from collaborative learning. However, there is a need to explore a specific framework, such as the flower framework, to conduct empirical studies and gain valuable insights into FL.

## 3. Preliminaries

This study presents a flower framework paradigm to implement various FL methods and conduct empirical studies. This section presents the preliminary results of the research presented in this paper.

### 3.1. Rationale of Using Flower Framework

The flower framework was chosen in this study because of its superior performance across several critical metrics when compared to other FL frameworks. A comparative evaluation of existing frameworks highlighted key factors such as communication overhead, bandwidth usage, round efficiency, latency, data leakage, security against attacks, and client anonymity. In terms of the communication complexity inherent in the collaborative learning process, the flower framework effectively managed the associated overhead. It incorporates mechanisms to optimize bandwidth utilization, thereby supporting large-scale collaboration among diverse parties with varied datasets and heterogeneous devices. Collaborative learning often involves multiple iterative rounds while assimilating data from different clients, which makes latency and round efficiency crucial considerations. The flower framework addresses these aspects through its robust underlying workflow and optimized mechanisms. Security concerns in collaborative learning, such as inference attacks leading to data leakage and other forms of cyber threats, are mitigated by the framework’s protective measures. Notably, it ensures client anonymity, which is a highly desirable feature in federated learning contexts where numerous participants are involved. The framework’s ability to keep participant identities confidential without compromising the integrity of the learning process enhances its suitability for sensitive applications.

### 3.2. The Flower Framework and Its Advantages

The flower framework represents a recent and flexible open-source implementation of FL that addresses many of the challenges noted in the literature:

- Modularity and Flexibility: Unlike some existing FL solutions, flower is designed to integrate various algorithms (e.g., FedAvg, FedProx, FedOpt) seamlessly, allowing researchers to experiment with different strategies within the same framework.
- Scalability: Flower is optimized for distributed environments and is capable of efficiently coordinating training across heterogeneous clients, reducing communication overhead and enhancing performance.
- Practical Deployment: Its user-friendly API and compatibility with various machine learning libraries make it an attractive choice for real-world applications in both IDS and fraud detection.

Furthermore, the flower framework supports proven methodologies to deliver effective solutions for organizations and domains engaged in federated learning, as shown in Table 2. Its adaptability allows the incorporation of diverse mechanisms based on real-time scenarios. Given the heterogeneity in various aspects of federated learning, the framework’s flexibility is instrumental in addressing runtime situations, particularly those related to privacy and security concerns. It provides robust client anonymity and safeguards sensitive information during the learning process.

As summarized in Table 2, we present an analysis of various federated learning frameworks, comparing critical aspects such as communication overhead, bandwidth usage, round efficiency, latency, data leakage, security against attacks, and client anonymity. Numerous frameworks offer different characteristics supporting FL mechanisms, and the flower framework was chosen for this study because of its intuitive design and adaptability to various contexts and runtime situations. Another significant factor influencing this choice is the capacity of the framework to uphold anonymity, prevent the leakage of sensitive data, and defend against potential security attacks. These features collectively make the flower framework an optimal choice for advancing research in federated learning and for implementing secure, efficient, and scalable collaborative learning solutions across diverse domains.

### 3.3. Relevance of IDS and Credit Card Fraud Detection

Fraud detection in the financial sector has evolved with the integration of machine learning techniques. Seminal works by [8,9] underscored the promise of supervised and unsupervised learning methods in identifying fraudulent transactions.

Despite these advancements, several challenges remain, as follows:

- Sensitive and Confidential Data: Financial institutions handle highly confidential data, and centralizing such information for model training increases the risk of data breaches.
- Data Imbalance: Fraudulent transactions are typically rare compared to legitimate ones, which complicates model training and often leads to suboptimal detection performance.
- Limited Generalizability: Models trained in a single institutional context may not perform well when deployed in different environments due to variations in transaction patterns.

These challenges call for an approach that can leverage distributed data while preserving privacy and improving generalization across institutions. IDS and credit card fraud detection can also benefit from FL, and FL is often used by organizations that share digital infrastructure. These organizations are vulnerable to cyber attacks, so protecting such infrastructure requires the ability to combine knowledge from multiple entities to protect sensitive data. FL allows entities to train ML models on their own data, and only model parameters are shared to create a global model, while raw data remains within each entity. This maintains privacy and client anonymity, therefore FL reduces data leakage and increases security, since sensitive information is not transferred. It enables detection of complex intrusion patterns by combining knowledge from multiple sources; thus, FL becomes a powerful tool in the field.

Since the global model aggregates attack vectors from different entities, it becomes more powerful and accurate in identifying cyber attacks, and this leads to enhanced real-time detection and response. FL is also useful in credit card fraud detection for financial institutions, because banks and credit card issuers generate huge amounts of transactional data used for fraud detection. However, these institutions often cannot share customer data due to privacy laws and ethical reasons, so FL allows each institution to train its own fraud detection model locally, and then combine insights into a global model that does not include individual transactional records. This allows detection of fraudulent patterns that may not be detectable in a single dataset, and frameworks such as flower enable FL by providing infrastructure to scale across varying data and computational environments.

## 4. Evidence and Procedure

This section describes the materials and methods used in the current research. This research focuses on the utility of the flower framework, enabling various FL algorithms to exploit its underlying benefits. The empirical study used various significantly proven algorithms with and without the flower framework to derive useful insights from the research.

### 4.1. Problem Statement

When there are numerous FL frameworks and algorithms available, exploring a specific framework such as flower is challenging. This is because of its inherent capability to address the complexities associated with collaborative learning, including privacy and security concerns, which are the focus of this research.

### 4.2. Evidence

Based on the object of this study, two datasets were carefully considered based on the feasibility of the case studies and their utility in the research of federated learning. These two benchmark datasets are widely used for intrusion detection and fraud detection. Intrusion detection is very important for any organization, including financial organizations. It is also important for financial organizations to deal with credit card fraud through a collaborative approach. Therefore, in this study, UNSW-NB15 was used for intrusion detection, while the credit card dataset was used for fraud detection. The use of the two benchmark datasets could help improve the diversity of data, leading to more generalizable observations possible with this research.

### 4.3. Procedure

This section describes the research methodology, and this study leverages the flower framework to utilize its features in the implementation of FL algorithms.

## 5. Comparative Analysis of Federated Learning Frameworks

### 5.1. Theoretical Comparison

To further establish the novelty of our work and justify our choice of the flower framework, we expanded our discussion to include a detailed theoretical comparison with other state-of-the-art FL solutions, such as TensorFlow Federated (TFF) and PySyft. This analysis examines key architectural and functional aspects:

- Modularity and Flexibility: Flower’s modular design allows seamless integration of various FL algorithms (e.g., FedAvg, FedProx, and FedOpt) while supporting heterogeneous client environments. This flexibility is essential for adapting to diverse data distributions and computational capabilities, which is particularly important in financial applications where data characteristics can vary significantly.
- Scalability and Communication Efficiency: The flower framework incorporates dynamic client selection and efficient model aggregation techniques that minimize communication overhead. Compared to TFF and PySyft, flower’s design better accommodates large-scale deployments, ensuring that the coordination among numerous clients remains efficient even under high-load conditions.
- Privacy and Security Features: In high-stakes sectors such as finance, protecting sensitive data is paramount. Flower’s architecture inherently supports privacy-preserving protocols by enabling local training on confidential data and secure model aggregation. This focus on privacy, along with its ease of integration with existing security measures, positions flower favorably against other frameworks that may require additional modifications to meet similar security standards.

### 5.2. Practical Evaluation

In addition to the theoretical insights, we conducted a set of experiments to compare the practical performance of the flower framework with TFF and PySyft. The experimental setup was standardized across all frameworks using the UNSW-NB15 and credit datasets, ensuring an apples-to-apples comparison based on the following metrics:

- Convergence Speed and Model Accuracy: We measured the number of communication rounds required for convergence and the final accuracy achieved by each framework. Our results demonstrated that while all frameworks maintained high accuracy levels (above 99%), the flower framework consistently reached convergence faster with a reduced number of communication rounds.
- Communication Overhead: In evaluation results show that flower incurs lower communication overhead by analyzing the volume of data exchanged during the federated training process. This efficiency is crucial in environments with limited bandwidth or when scaling to larger clients.
- Ease of Deployment and Integration: Practical deployment considerations were also evaluated, such as the ease of integrating with existing machine learning pipelines and the availability of customization options.

Flower’s intuitive API and flexible architecture enabled rapid prototyping and deployment, which is particularly advantageous for organizations with varying technical resources. Table 3 focuses on the deep learning models used in prior studies and compares them with the enhanced CNN model implemented in this study. Table 4 shows that the flower framework outperforms other federated learning solutions by offering faster convergence and improved communication efficiency. Its secure, privacy-preserving design makes it ideal for sensitive applications like finance and IDS. Additionally, flower’s ease of deployment, scalability, and low overhead provide a strong balance between theoretical robustness and practical real-world applicability. Our evaluation, which tests several FL algorithms with and without flower, confirms its effectiveness in enhancing collaborative learning. Flower is generic and integrates with various datasets, models, and devices for real-world FL; therefore, it allows integration with TensorFlow 2.11.0, PyTorch 1.13.1, and Scikit-learn 1.2.2 for various models and algorithms. Flower provides the flexibility to handle non-IID data, which is the norm in FL problems such as intrusion detection, fraud detection, and many other real-world problems where data is highly non-IID between clients; thus, flower supports aggregation strategies such as FedAvg, FedProx, and FedOpt to handle non-IID data efficiently. Flower scales to many clients including low-end devices without loss in performance, and flower’s small clients and efficient communication make it suitable for low-power networks such as IoT. Flower ensures data privacy by training on the client and sharing only the model updates with the server because with secure aggregation, privacy is guaranteed and flower can be applied to sensitive fields such as finance and security. This paper used flower to evaluate FL algorithms on the UNSW-NB15 intrusion dataset and the credit card fraud dataset, so it demonstrates the versatility of flower and showcases how flower addresses the challenges of collaborative learning, including non-IID data, faster training, and lower communication. By leveraging flower in this paper, we can explore how it can enhance the performance and scalability of FL algorithms in practical scenarios, thus making it a versatile tool for various domains.

To guarantee normality, numerical features were first identified, logarithmic transformation was applied using np.log1p(), and the distributions were then shown using histograms. Correlation analysis was performed using df.corr() to calculate the correlation matrix and sns.heatmap() to visualize it on a heatmap. Outliers were identified using the interquartile range (IQR) method, calculating Q1 (25th percentile) and Q3 (75th percentile) for each numeric feature, and identifying outliers as data points falling below Q1 − 1.5 × IQR or above Q3 + 1.5 × IQR. Numeric features were scaled to a range of 0 to 1 using MinMaxScaler() and categorical features (state, service, and proto) were scaled using LabelEncoder().

**Figure 1:**
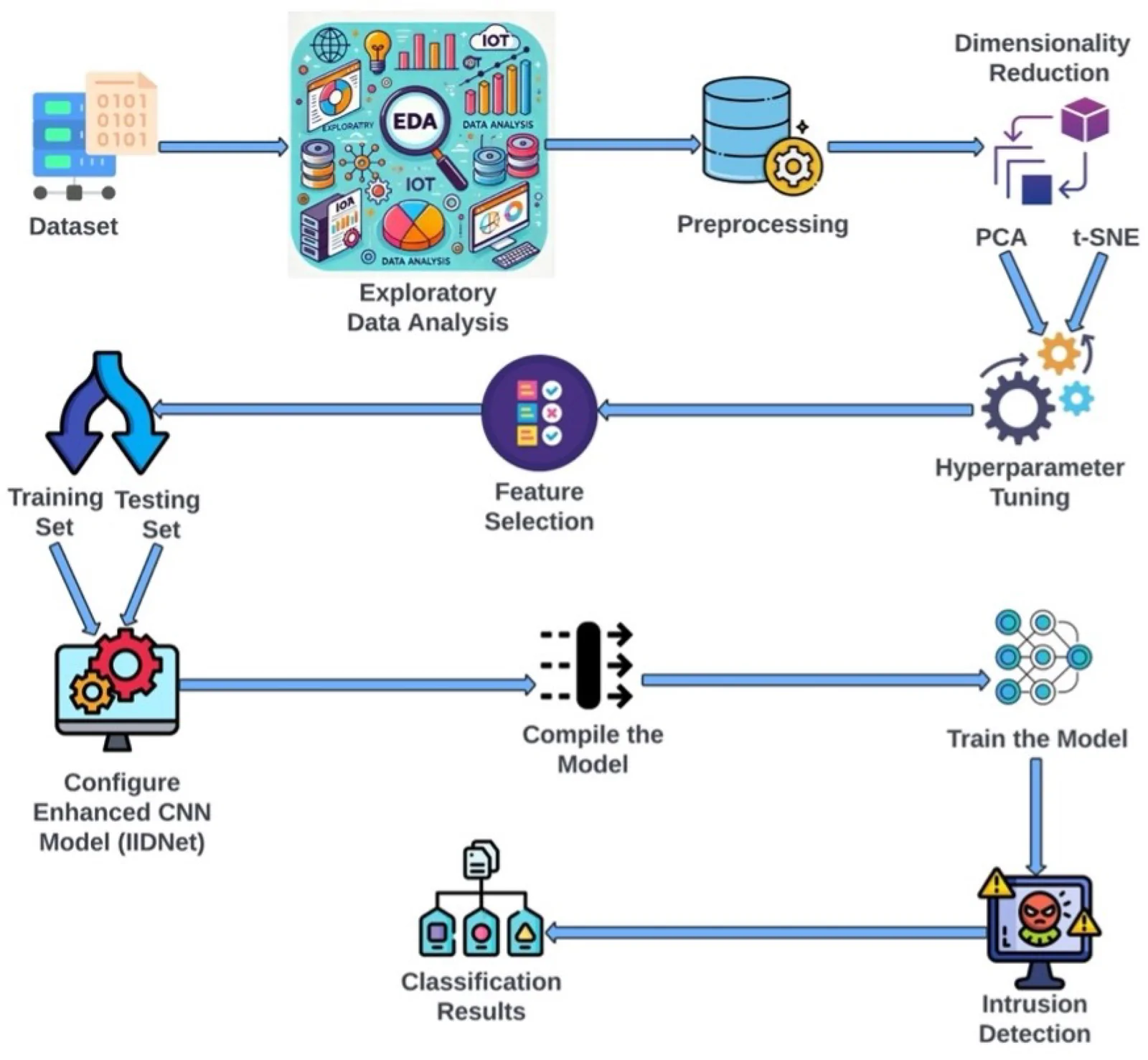
Preprocessing of data [56].

Principal component analysis (PCA) was applied to reduce dimensionality while preserving variance, fitting PCA on the combined features, and transforming the data into principal components, with the explained variance ratio for each principal component visualized. In this regard, class balancing was achieved by oversampling the minority class (malicious) using the sample() method. Dimensionality reduction using t-SNE (t-distributed stochastic neighbor embedding) was applied to visualize high-dimensional data in 2D space, plotting the transformed data points to show the separation between normal and malicious classes. Feature importance was assessed using an XGBoost model, plotting feature importance with xgb.plot_importance(), and selecting important features based on a defined threshold for further analysis. The final dataset was prepared with selected important features and labels, separated into training and testing sets for model development and validation. The intrusion detection model implemented in this study is shown in Figure 1.

**Figure 2:**
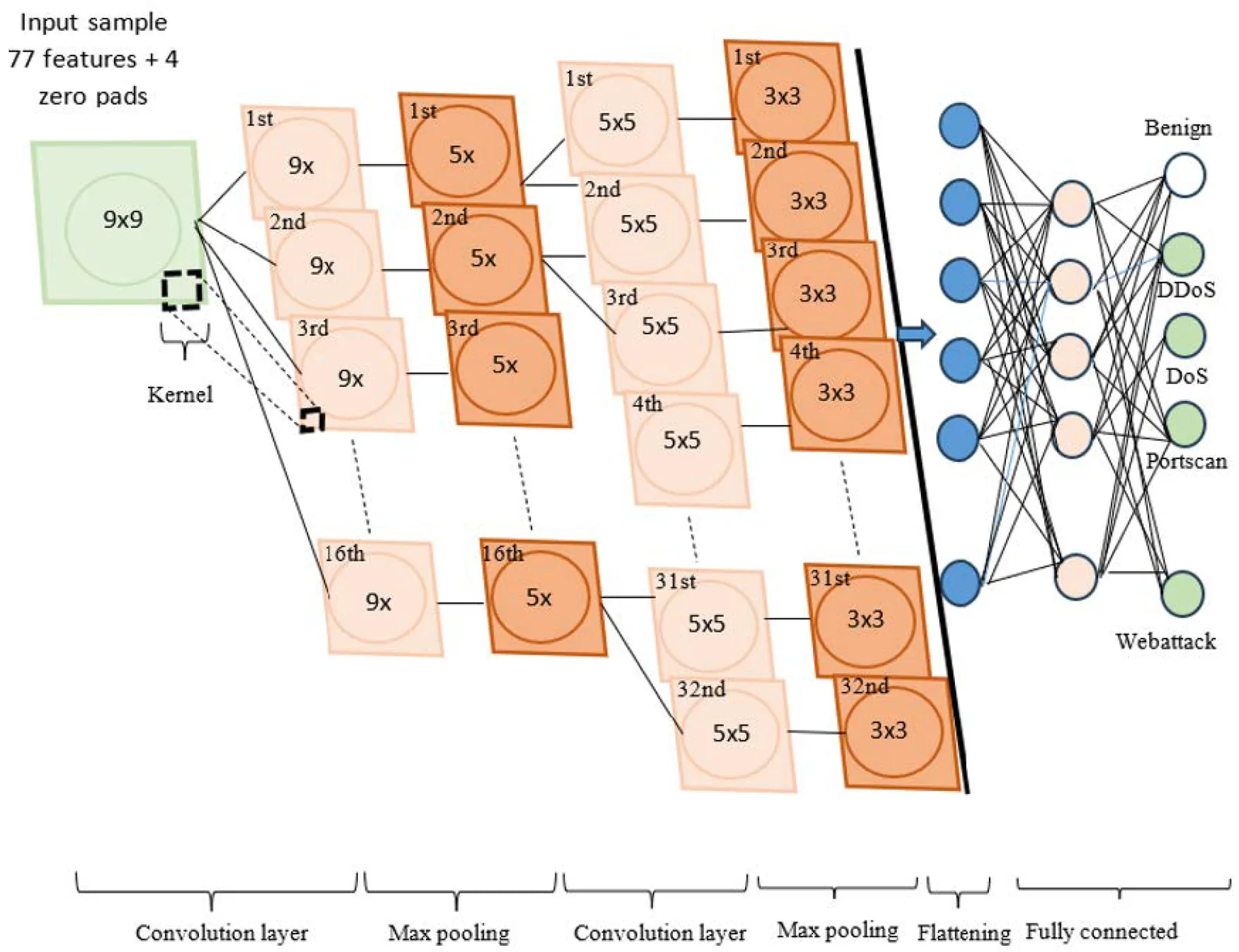
Proposed architecture of enhanced CNN known as IIDNet (intelligent intrusion detection network) [56].

A convolutional neural network (CNN) was used to classify network traffic and credit card transactions as shown in Figure 2. The CNN architecture included input layers to receive preprocessed data, convolutional layers (Conv1D/Conv2D) with filters (e.g., 32, 64) and kernel sizes (e.g., 3 × 3, 5 × 5) to capture spatial features, and ReLU activation functions to introduce non-linearity. MaxPooling layers were used to reduce the spatial dimensions and computational complexity. Dropout was also applied during training (with a dropout rate of 0.25 after the pooling stages and 0.5 before the output layer) as a standard regularization technique to mitigate overfitting; these regularization layers are omitted from the simplified architecture diagram in Figure 2 and the layer summary in Table 5 purely for readability, but they were part of the model used during training. Dense layers (e.g., 128 and, 64 units) performed classification based on features extracted by convolutional layers, with ReLU activation functions in hidden dense layers and softmax or sigmoid activation functions in the output layer for multi-class or binary classification, respectively. It is worth noting that the input representation is a 9 × 9 matrix containing 77 feature values (with four zero-pads to complete the grid). The UNSW-NB15 dataset originally provides 42 features, and the expansion to 77 occurs because categorical attributes (such as proto, service, and state) are one-hot encoded during preprocessing, which is a standard feature engineering step that converts each categorical attribute into several binary columns.

The hyperparameters and training setup included a batch size of 32, 50 global communication rounds (with a small number of local epochs per round at each client), a learning rate of 0.001, and the use of the Adam optimizer both on the client side and, where applicable (for example, in the server-side update step of FedOpt), on the server side. The loss functions used were binary cross-entropy for binary classification tasks (the credit card fraud detection case, where the label indicates fraudulent or legitimate transactions) and categorical cross-entropy for multi-class classification tasks (the UNSW-NB15 case, where the label denotes the attack category as reflected in Table 5). Early stopping with a patience of five epochs was employed to prevent overfitting by monitoring the validation loss, and model checkpointing was used to save the best model based on the validation loss. For FedProx, the proximal regularization coefficient *μ* was set to a small constant value (0.01), consistent with values commonly reported in the literature for similarly sized models and datasets. For our federated setup, the training data were partitioned horizontally across ten clients using a non-IID split, so that each client held a subset of the samples while all clients shared the same feature space; in every round, all ten clients participated (i.e., *K* = 10) rather than a randomly sampled subset, in order to keep the experimental comparison across algorithms and rounds stable. Table 5 presents the layers involved in the enhanced CNN model. Further details on code availability, repository organization, the experimental environment, and reproducibility are provided in the Supplementary Materials.

**Figure 3:**
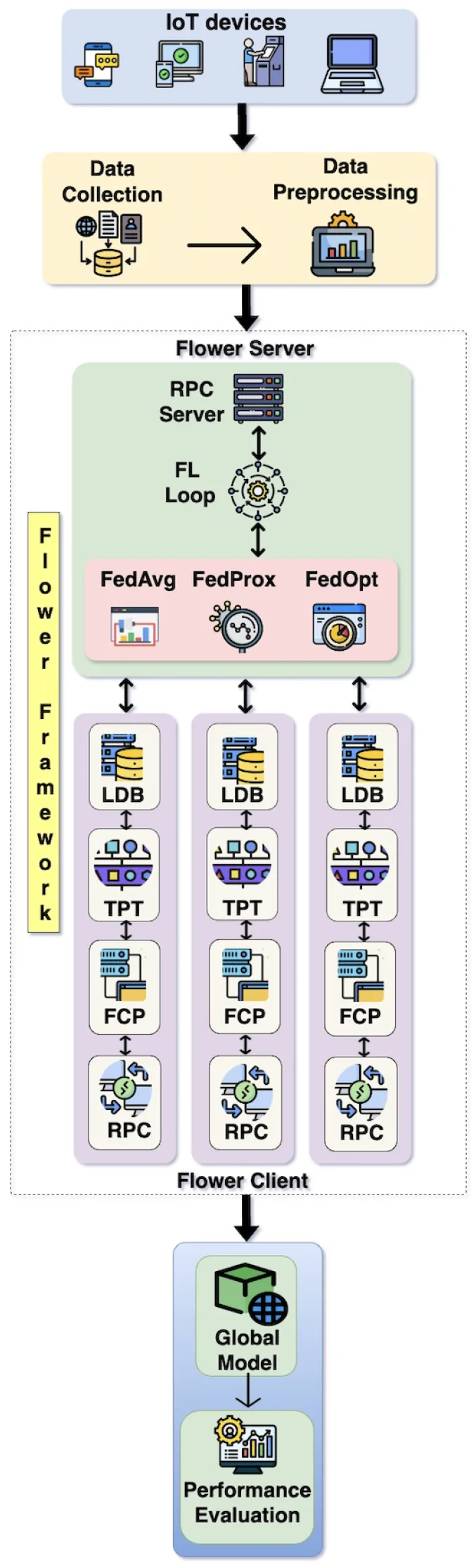
Architecture of proposed enhanced CNN known as IIDNet (intelligent intrusion detection network).

Figure 3 depicts the architecture of IIDNet, which is an enhanced convolutional neural network specifically designed for intelligent intrusion detection. To integrate the federated learning algorithms FedAvg, FedProx, and FedOpt within the flower framework using IIDNet, each client in the federated learning environment utilizes IIDNet as their local model for intrusion detection tasks. The flower framework facilitates communication between clients and the central server, orchestrating the federated learning process. In each training round, clients used their local datasets to train IIDNet, updating the model’s weights based on their unique data. After local training, clients send their updated model weights to the central server. The server then applies the different aggregation algorithms:

- FedAvg: Aggregates by averaging the model weights from all clients to create an updated global model.
- FedProx: Incorporates a proximal term in the local objective function to address data heterogeneity before aggregation and to enhance stability during training.
- FedOpt: Employs advanced optimization techniques on the server side to improve convergence and overall model performance.

In the flower framework setup, the clients are equipped with local data preparation and processing tools, including remote procedure calls (RPC), flower client Python (FCP), training pipeline TensorFlow (TPT), and local databases (LDB). Each client independently prepares its data locally and participates in the training process. The FL loop is managed by the flower server, and it uses the FedAvg, FedProx, and FedOpt algorithms to train local models on client data. The flower server receives the locally learned model weights from the clients at the end of each training cycle and combines them to update the global model. By repeating this procedure 50 times, the global model was continuously improved using a variety of client datasets. A central server oversees the federated learning procedure in the conventional federated learning configuration, which excludes the flower framework. Customers use the FedAvg, FedProx, and FedOpt algorithms to train their local models independently. Akin to the flower configuration, following every training cycle, the local model weights are communicated to the central server, which then aggregates them to refresh the global model. The clients participated in 50 rounds of this iterative process.

The proposed architecture includes unique privacy-preserving federated learning models and processes. ROC, AUC, F1 score, accuracy, precision, recall, and other performance measures were used to assess the final global model produced by the flower and conventional setups. To improve security and privacy for intrusion and fraud detection, this review evaluates the effectiveness of federated learning systems. The methodology ensures that the global model benefits from the diverse data distributed across all clients, while maintaining data privacy and security.

### 5.3. Algorithm Design

To address the issues of privacy and efficiency in collaborative machine learning, we propose a novel framework that leverages FL to maintain data privacy in distributed networks. We explore the effectiveness of three FL methods—FedAvg, FedProx, and FedOpt—by applying them to the credit card and UNSW NB-15 datasets within federated flower environments. Before defining algorithms using the flower framework paradigm, we discuss the rational design considerations of federated learning.

i.    Federated Transfer Learning: FL can enable federated transfer learning, in which knowledge from one task or domain can be transferred to another without sharing raw data. This allows models to be trained on specialized tasks by leveraging pre-trained models from different institutions or domains while maintaining privacy.
ii.   Resource Efficiency: With respect to resource efficiency, design decisions are made to exploit the flower framework’s many existing full algorithms, which benefit from the framework’s inherent mechanisms in terms of using resources more efficiently.
iii.  Edge Intelligence: FL with the flower framework can enable various clients to be used in the learning process. Because the framework supports various types of devices and heterogeneity in communication protocols and locally available datasets, it enables the use of edge computing devices to ensure edge intelligence in the collaborative learning process.
iv.   Personalized Models: The framework supports personalized machine learning models based on the client’s local datasets. These models can provide the required knowledge of data analytics that the FL system can collaboratively use without sharing data.
v.    Increased Trust Among Participants: The collaborative learning process provides an environment in which various clients can participate under the knowledge required for decision-making. Moreover, by using the flower framework, various security and privacy mechanisms besides client anonymity can be in place, which will benefit increased trust among various participants involved in the system.
vi.   Reduced Legal Risks: The collaborative learning process, with the help of the flower framework, enables various clients to participate seamlessly in the learning process without compromising privacy and security. Since the flower framework comes with built-in functionalities pertaining to security and inference attacks, besides providing client anonymization, there is less scope for legal risks.
vii.  Enhanced Resilience to Data Poisoning: The underlying phenomenon of FL involves mechanisms to deal with collaborative learning without expecting clients to share their local data. Moreover, the proposed research exploits the flower framework, which has mechanisms for dealing with data poisoning.
viii. Improved Fairness and Inclusivity: In process of collaborative learning, various clients are allowed in an inclusive fashion to participate in learning and acquire the required knowledge to help in decision-making. The use of the flower framework also improves fairness in supporting diversified clients and heterogeneous environments.

#### 5.3.1. FedAvg Algorithm

By working with many clients to repeatedly collaboratively refine a global model, the FedAvg Algorithm 1 is intended to support federated learning. The global model weights $w_{0}$ were initialized first. Subsequently, the procedure was performed through *T* communication cycles. A subset $S_{t}$ of *K* clients is chosen at random to participate in each round. Using their own data, each client in the subset performed local training after downloading the most recent global model weights, $w_{t}$ , from the central server. More precisely, every client performs *E* epochs of stochastic gradient descent (SGD) to update the model weights, which yields local model weights $w_{t+1}^{k}$ .
**Algorithm 1** FedAvg: federated averaging algorithm.
1:**Input:** Number of rounds *T*, number of clients *K*, learning rate η
2:Initialize global model weights $w_{0}$
3:**for** each round t=1,…,T **do**4:     Sample a subset $S_{t}$ of *K* clients5:     **for** each client $k\in S_{t}$ in parallel **do**6:           Download global model weights $w_{t}$
7:           Compute local model weights $w_{t+1}^{k}$ by performing *E* epochs of SGD on local data8:     **end for**9:     Aggregate local models: $w_{t+1}=\frac{1}{K}\sum_{k\in S_{t}}w_{t+1}^{k}$
10:**end for**11:**Return:** final global model weights $w_{T}$



Similar to the first algorithm, the updated weights from each participating client were combined by the central server following local training. The new global model weights were derived by averaging the local model weights using the following formula:

(1) $$w_{t+1}=\frac{1}{K}\sum_{k\in S_{t}}w_{t+1}^{k}$$

For every *T* cycle, this cyclical process of local training followed by global aggregation was performed. The ultimate global model weights, $w_{T}$ , are returned once the designated number of rounds is completed. With this approach, data privacy is preserved by keeping raw data localized on client devices, whereas the global model benefits from the diversity of data possessed by all clients.

#### 5.3.2. FedProx Algorithm

Specifically designed to address the difficulties posed by diverse data and different computing settings across users, the FedProx Algorithm 2 is an improvement of the FedAvg method. The global model weights $w_{0}$ were initialized first. Over *T* communication cycles, the training procedure iterates, with a random selection of $S_{t}$ clients from a set of *K* clients to participate in each round. The current global model weights, $w_{t}$ , are downloaded from each of these chosen clients from the main server. Next, by utilizing the proximal stochastic gradient descent (SGD), clients engage in local training. They specifically updated the local model weights $w_{t+1}^{k}$ by minimizing a modified objective function as follows:

(2) $$L\left(w\right)+\frac{\mu }{2}{∥w-w_{t}∥}^{2}$$

Here, the proximal term, $\frac{\mu }{2}{∥w-w_{t}∥}^{2}$ , penalizes the departure of local weights from global weights. The local loss function is represented as L(w) . To stabilize the training process and reduce the effects of client heterogeneity, the proximal term, μ is essential. As in Algorithm 2, after completing *E* epochs of proximal SGD, clients send their updated local model weights $w_{t+1}^{k}$ back to the central server. The server aggregates these local models by averaging them to create new global model weights $w_{t+1}$ , using Equation (1).

This cycle of local training and global aggregation was repeated for the entire duration of *T* rounds. Once all the rounds are completed, the final global model weights $w_{T}$ are returned. By incorporating the proximal term in the objective function, FedProx ensures greater stability and robustness in the model, making it particularly well-suited for real-world federated learning applications where clients exhibit significant differences in data distributions and computational capabilities.
**Algorithm 2** FedProx: federated proximal algorithm.
1:**Input:** Number of rounds *T*, number of clients *K*, learning rate η , proximal term μ
2:Initialize global model weights $w_{0}$
3:**for** each round t=1,…,T **do**4:      Sample a subset $S_{t}$ of *K* clients5:      **for** each client $k\in S_{t}$ in parallel **do**6:            Download global model weights $w_{t}$
7:            Compute local model weights $w_{t+1}^{k}$ by performing *E* epochs of proximal SGD on local data, minimizing the objective: $L\left(w\right)+\frac{\mu }{2}{∥w-w_{t}∥}^{2}$
8:       **end for**9:       Aggregate local models: $w_{t+1}=\frac{1}{K}\sum_{k\in S_{t}}w_{t+1}^{k}$
10:**end for**11:**Return:** final global model weights $w_{T}$



#### 5.3.3. FedOpt Algorithm

This algorithm is defined to have enhanced effectiveness in the process of model training and adaptation to runtime situations associated with various clients with different data modalities. It has mechanisms to exploit the environment, including the server and clients, in a collaborative fashion, where the model weights are globally utilized when the local models and local datasets are kept intact. This algorithm exploits its mechanisms, where the local training derives knowledge which is used in the global learning part. In this algorithm, each client can have a different model of choice with desired optimizations and configurations, but the weights gained should be sent to the server to have a collaborative learning process.

As in Algorithm 3, once local training is completed, the clients send their updated local model weights back to the central server. The server then computes the gradient $g_{t}$ as the average difference between the global model weights $w_{t}$ and the local model weights $w_{t+1}^{k}$ across all participating clients, using the formula

(3) $$g_{t}=\frac{1}{K}\sum_{k\in S_{t}}\left(w_{t}-w_{t+1}^{k}\right)$$

This gradient is used to update the state to $s_{t+1}$ through the server optimizer $O_{s}$ :

(4) $$s_{t+1}=O_{s}.\mathrm{update}\left(s_{t},g_{t}\right)$$

The global model weights are then updated using the server optimizer’s step function, resulting in

(5) $$w_{t+1}=w_{t}-O_{s}.\mathrm{step}\left(s_{t+1}\right)$$

This process was repeated for all *T* rounds. The final global model weights $w_{T}$ are returned at the end of the training process. This iterative process ensures that the global model progressively improves by leveraging both local client updates and sophisticated server-side optimization techniques.
**Algorithm 3** FedOpt: federated optimization algorithm.
1:**Input:** Number of rounds *T*, number of clients *K*, server optimizer $O_{s}$ , client optimizer $O_{c}$
2:Initialize global model weights $w_{0}$
3:Initialize server optimizer state $s_{0}$
4:**for** each round t=1,…,T **do**5:      Sample a subset $S_{t}$ of *K* clients6:      **for** each client $k\in S_{t}$ in parallel **do**7:            Download global model weights $w_{t}$
8:            Compute local model weights $w_{t+1}^{k}$ by performing *E* epochs of $O_{c}$ on local data9:      **end for**10:     Compute gradient $g_{t}=\frac{1}{K}\sum_{k\in S_{t}}\left(w_{t}-w_{t+1}^{k}\right)$
11:     Update server optimizer state $s_{t+1}=O_{s}.\mathrm{update}\left(s_{t},g_{t}\right)$
12:     Update the global model weights $w_{t+1}=w_{t}-O_{s}.\mathrm{step}\left(s_{t+1}\right)$
13:**end for**14:**Return:** final global model weights $w_{T}$



### 5.4. Utility of the Proposed System

**Figure 4:**
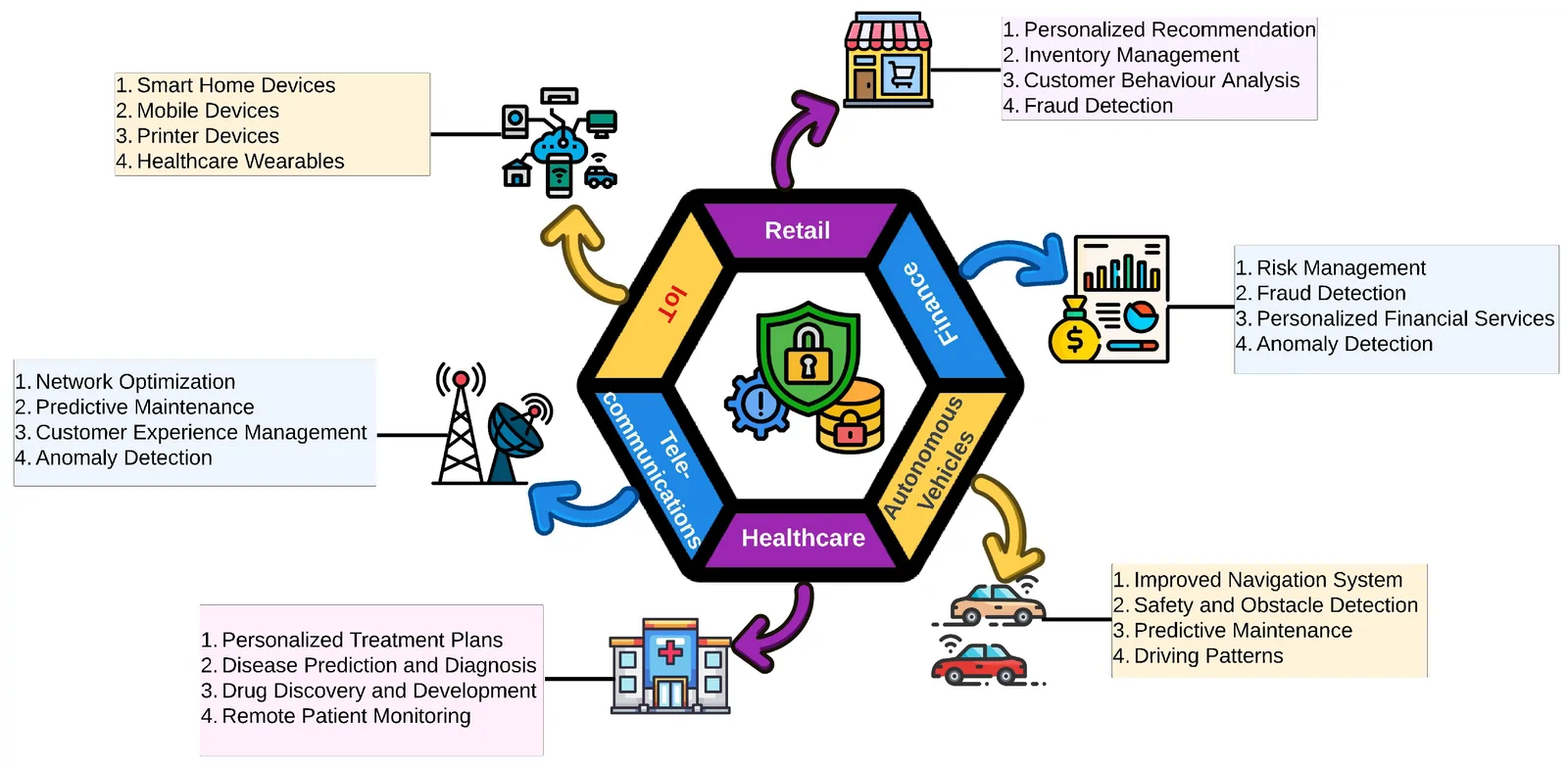
Applications of the proposed approach.

FL with a flower framework has a wide array of applications across various industries, as shown in Figure 4, leveraging the ability to train models collaboratively without sharing raw data. The collaborative learning process generally avoids privacy leakage and enables clients to have customized models and optimizations towards having greater freedom but still participates in the collaborative learning process to derive the required actionable knowledge. The framework enables the participation of various clients with complete anonymity and privacy to acquire knowledge from not only local data but also data from various other clients. That is, the power of federated learning, where knowledge is objectively collaborated and used to make informed decisions. This kind of approach is very useful in domains such as finance, where data security and privacy are given paramount importance and also help in the derivation of new knowledge not only from local data but also from other related organizations.

The proposed federated learning framework, leveraging the flower framework, can be deployed in real-world IoT-enabled digital infrastructures by enabling decentralized model training directly on IoT devices. In this setup, each IoT device (or group of devices) trains a local machine learning model using its own data, without sharing the raw data with a central server. Local model updates are then aggregated to form a global model, ensuring that sensitive data remain on the devices where they are generated. Deploying the system in this manner offers several benefits over the traditional centralized approaches. First, it enhances data privacy and security by keeping raw data localized, which is crucial for IoT devices, which often handle sensitive information. Second, it reduces bandwidth consumption and network congestion because only model updates, which are not all datasets, are transmitted. This is particularly advantageous in IoT networks where devices may have limited connectivity or bandwidth. Additionally, the framework improves scalability and resilience; new devices can join the federated network without significant changes to the infrastructure, and the system is less vulnerable to single points of failure.

Financial organizations may train models jointly on a larger decentralized dataset, which increases fraud detection. It also allows tailored financial services by securely analyzing customer data from many platforms. Moreover, FL facilitates the early detection of fraudulent activity by observing odd trends in financial transactions. FL improves route planning and traffic management for autonomous cars by training navigation algorithms on the data gathered from several vehicles. By allowing cars to learn from one another’s mistakes and strengthening their obstacle recognition and avoidance systems, safety features can be improved. Analyzing the data improves predictive maintenance.

Dependability can be enhanced by improving the availability and performance data of a fleet of cars, and therefore, this improvement can lead to better overall operations. Better autonomous driving algorithms can be achieved by using FL’s assistance in comprehending and forecasting driving behaviors. Through privacy-preserving model training on patient data from several institutions, FL makes it possible to create customized treatment plans in the healthcare industry. Using securely stored patient data, it enables medical facilities to work together to develop more precise models for illness diagnosis and prediction. By enabling continuous learning from patient data collected from various devices, FL improves remote patient monitoring and speeds up drug discovery by enabling pharmaceutical companies to train models on data from multiple sources without disclosing proprietary information.

By learning from data spread across several network nodes, FL aids in the optimization of network performance in telecommunications. By evaluating data from several sites, it forecasts equipment failures and maintenance requirements and enhances customer service by analyzing user data to find and fix problems early. Additionally, FL detects anomalies in network traffic, helping to identify and mitigate security threats. In Internet of Things (IoT) applications, FL enables smart home devices to learn and improve their functions by collaborating on data without sharing personal information. Mobile applications can enhance user experience by learning from usage patterns across devices while preserving user privacy. FL optimizes printer performance and predicts maintenance requirements by learning from usage data across multiple devices. Finally, FL improves the functionality of healthcare wearables by securely training health data from various users, thereby enhancing health monitoring and recommendation.

### 5.5. Evaluation of Methodology

The performance of the federated learning models was evaluated using the following metrics:

- Accuracy:
  - Definition: The ratio of correctly predicted instances to the total instances in the dataset.
  - Formula:
(6) $$\mathrm{Accuracy}=\frac{TP+TN}{TP+TN+FP+FN}$$
  - Importance: Measures the overall correctness of the model’s predictions.
- Loss:
  - Definition: A measure of how well the model’s predictions match the true labels. It quantifies the error made by the model during training and evaluation.
  - Formula:
(7) $$\mathrm{Loss}=-\frac{1}{N}\sum_{i=1}^{N}\left[y_{i}\log\left({\hat{y}}_{i}\right)+\left(1-y_{i}\right)\log\left(1-{\hat{y}}_{i}\right)\right]$$
    where *N* is the number of samples, $y_{i}$ is the true label, and ${\hat{y}}_{i}$ is the predicted probability.
  - Importance: Helps in understanding the model’s learning progress and convergence.
- Recall:
  - Definition: The ratio of correctly predicted positive instances to all actual positive instances.
  - Formula:
(8) $$\mathrm{Recall}=\frac{\mathrm{TP}}{\mathrm{TP}+\mathrm{FN}}$$
  - Importance: Measures the model’s ability to identify all relevant instances.
- Precision:
  - Definition: The ratio of correctly predicted positive instances to all predicted positive instances.
  - Formula:
(9) $$\mathrm{Precision}=\frac{\mathrm{TP}}{\mathrm{TP}+\mathrm{FP}}$$
  - Importance: Measures the accuracy of the positive predictions made by the model.
- F1 Score:
  - Definition: The harmonic mean of precision and recall, providing a balance between the two metrics.
  - Formula:
(10) $$F1\;\mathrm{Score}=\frac{2\cdot \mathrm{Precision}\cdot \mathrm{Recall}}{\mathrm{Precision}+\mathrm{Recall}}$$
  - Importance: Helpful in assessing models on unbalanced datasets when recall and accuracy are equally crucial.
- AUC (Area Under the ROC Curve):
  - Definition: The area under the receiver operating characteristic (ROC) curve was used to plot the genuine positive rate (recall) against the false-positive rate.
  - Formula:
(11) $$\mathrm{AUC}=\int_{0}^{1}\mathrm{TPR}\left(FPR\right)\;d\left(FPR\right)$$
    where TPR is the true positive rate and FPR is the false positive rate.
  - Importance: It provides an all-inclusive measure of the model’s performance across all categorization criteria.

## 6. Results and Discussion

We used the UNSW NB-15 [57] and credit card [58,59,60,61,62,63,64,65,66] datasets to assess the efficacy of the flower framework on three federated learning algorithms: FedAvg, FedProx, and FedOpt. Recall, accuracy, precision, F1 score, AUC, and recall are only a few of the measures that show significant gains in performance. It is worth clarifying that the numerical results reported here are not directly comparable with those of our prior centralized work [56] that used the same enhanced CNN backbone, as the training paradigm (federated vs. centralized), data partitioning, and optimization dynamics differ; any improvements observed here should therefore be interpreted in the context of the federated setting rather than as a head-to-head improvement over the earlier centralized model. High-performance computer resources were accessible using Google Colab, and this was used in the experimental setting. The runtime type utilized was an NVIDIA L4 GPU, which provides the improved computing power required for the deep learning model training. Linux is an operating system provided by Google Colab for software environments. Model training, assessment, and data preparation were made easier using Python 3.10 as the main programming language. Scikit-learn 1.2.2 was used for data preprocessing, model evaluation, and metric calculation. Pandas 2.2.3 and NumPy 2.0.2 were used for data manipulation and numerical computations, and TensorFlow 2.18.0 and Keras 3.8.0 were used to create and train convolutional neural network (CNN) models. Seaborn 0.13.2 and Matplotlib 3.10.0 were used for data visualization and plot of results. Furthermore, federated learning algorithms, such as FedAvg, FedProx, and FedOpt, were made easier to use by implementing the flower framework. The importance of the flower framework was examined through an analysis of the actual findings obtained both with and without its application.

### 6.1. Results With and Without Framework and UNSW-NB15 Dataset for Ten Rounds

**Figure 5:**
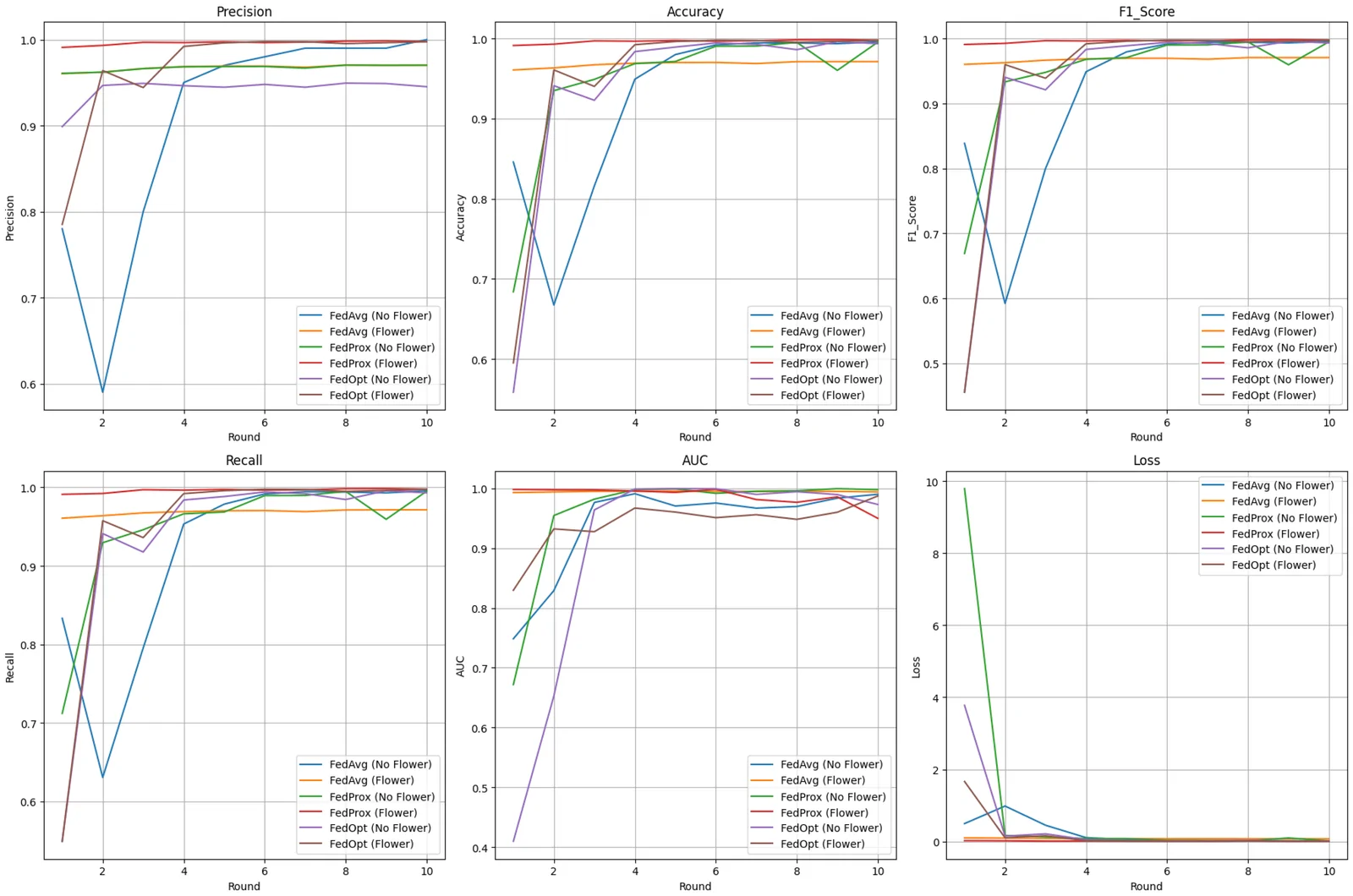
Performance comparison with and without flower framework using UNSW-NB15 dataset for 10 rounds.

The results presented in Figure 5 illustrate the substantial impact of employing the flower framework on the effectiveness of federated learning algorithms when deployed on the UNSW-NB15 dataset. When evaluating the algorithms without the flower framework, the initial rounds showed considerable variability in performance. For instance, FedAvg starts with an accuracy of 84.58% and gradually improves to 99.34% by the 10th round. FedProx, on the other hand, began with a lower accuracy of 68.39%, although it also increased to a high of 99.53% by the 10th round. FedOpt starts with the lowest accuracy of 55.87%, highlighting its initial instability and difficulty in adapting without the flower framework, although it reached 99.52% by the final round. These results suggest that while all three algorithms can eventually achieve high accuracy, their performance without the flower framework is inconsistent, especially in the initial rounds.

The initiation of the flower framework resulted in marked improvements in all metrics and algorithms. FedAvg, which had already performed relatively well without the framework, saw an immediate boost, starting with 96.06% accuracy in the first round and reaching a near-perfect accuracy of 99.77% by the 10th round. FedProx and FedOpt experienced even more significant improvements. FedProx’s initial accuracy jumps to 99.11%, and FedOpt, which had the weakest performance without the flower framework, significantly improved to 99.14% in the first round. Both algorithms then stabilized at approximately 99.77% and 99.83% accuracy, respectively, by the final round.

In addition to accuracy, other metrics such as precision, F1 score, and recall follow similar trends. Without the flower framework, FedAvg and FedProx show a steady increase in these metrics, whereas FedOpt lags, particularly in the initial rounds. However, with the flower framework, all three algorithms not only achieved higher precision, F1 scores, and recall values, but also demonstrated more consistency across rounds. This consistency is crucial for intrusion-detection tasks where reliability is of paramount importance. Overall, the findings clearly demonstrate that the flower framework significantly enhances the performance and stability of federated learning algorithms in the context of intrusion detection using the UNSW-NB15 dataset. The results suggest that integrating the flower framework could be a key factor in achieving high accuracy, precision, and robustness, thereby making it an essential component for practical deployment in securing digital infrastructures.

### 6.2. Results With and Without Framework and Credit Card Dataset for Ten Rounds

**Figure 6:**
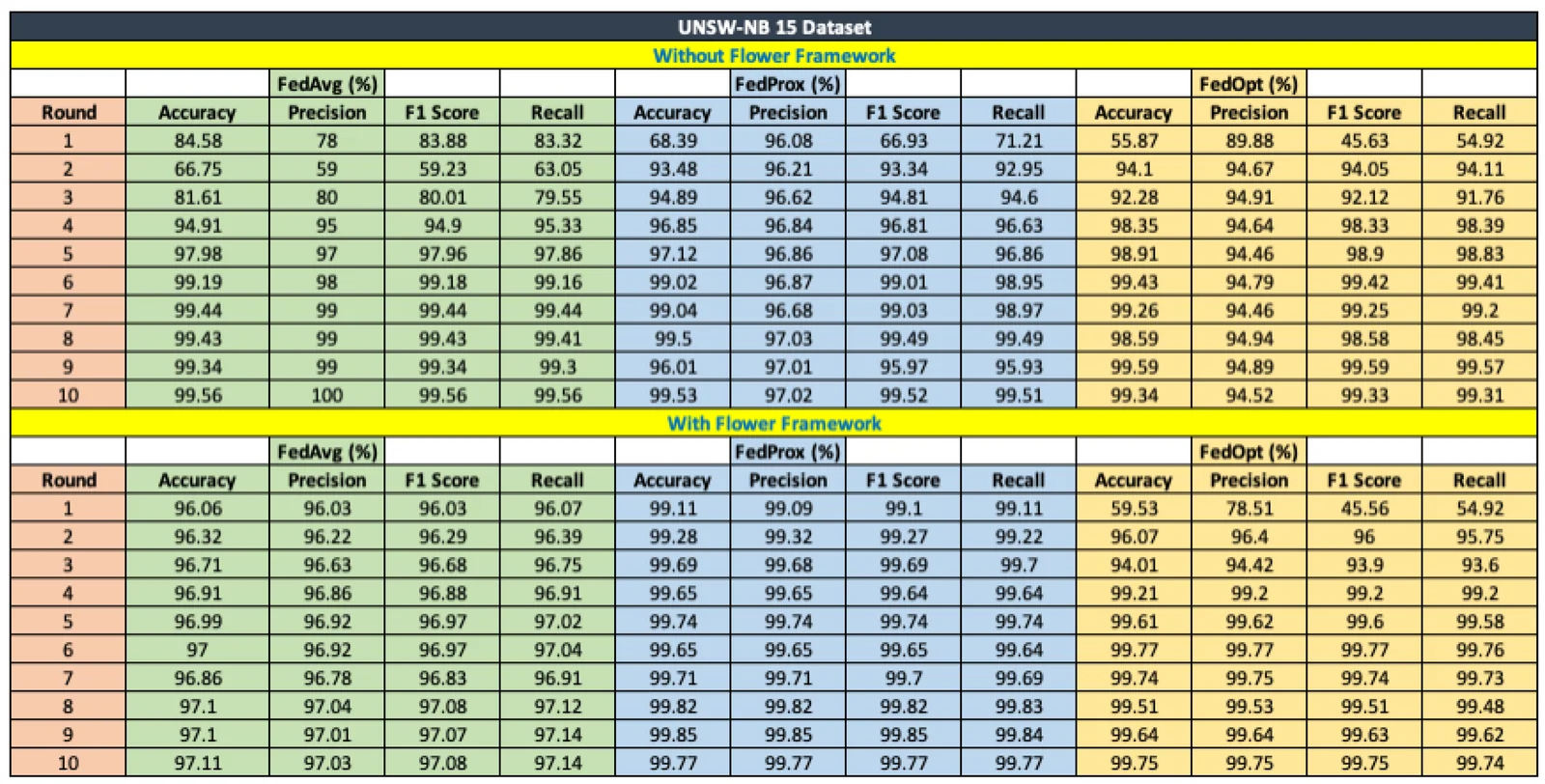
Results with and without flower framework using UNSW-NB15 dataset for 10 rounds.

**Figure 7:**
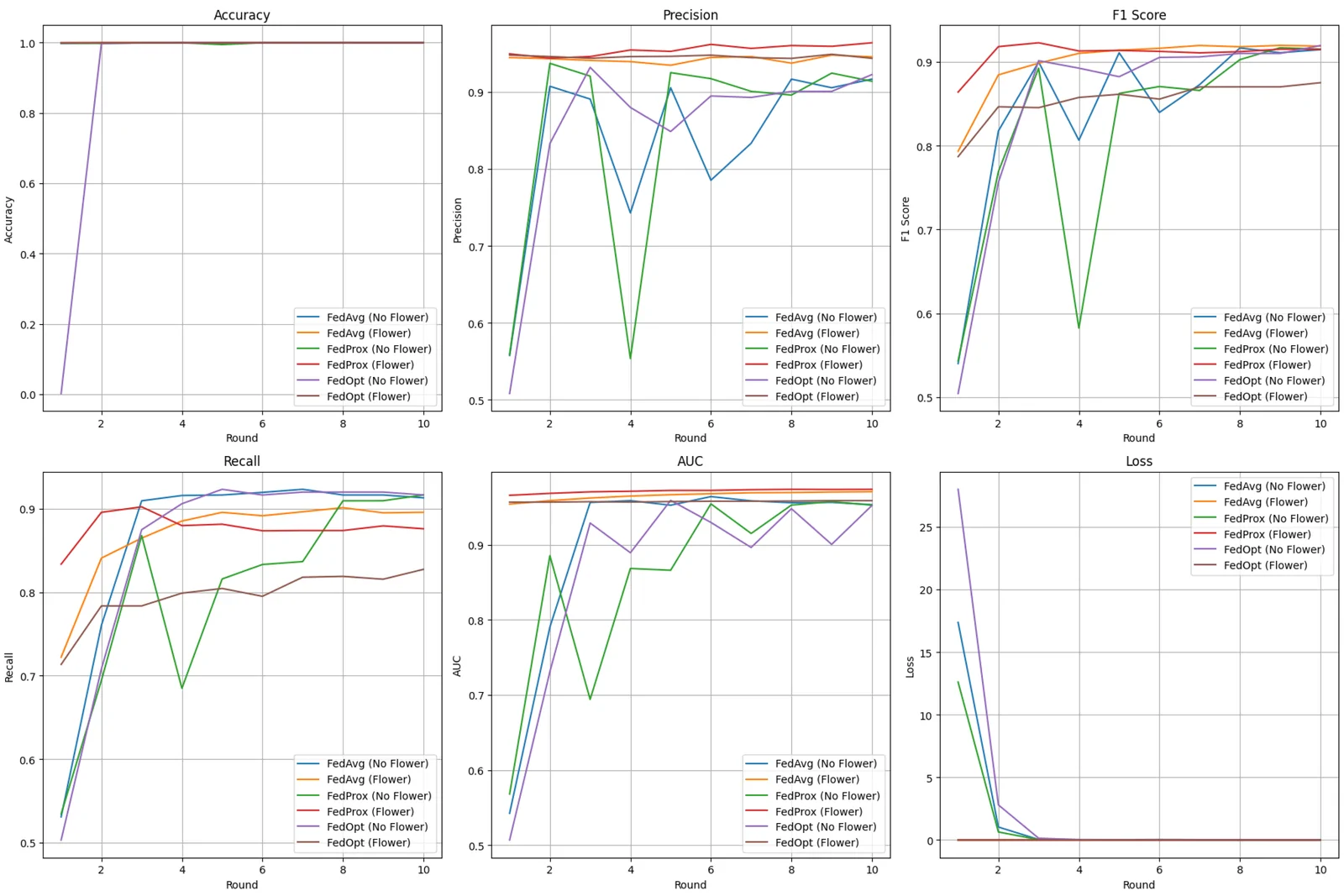
Performance comparison with and without flower framework using credit card dataset for 10 rounds.

As shown in Figure 6, the federated learning algorithms (FedAvg, FedProx, and FedOpt) have a large accuracy improvement when the Flower framework is applied in the credit data, and Figure 7 demonstrates the large gap in accuracy with and without the Flower framework after 10 rounds in the credit card data, which indicates the advantages of using the Flower framework for federated learning algorithms. Without the flower framework, FedAvg shows high accuracy from the start, reaching 99.94% in the first round and maintaining it throughout all rounds. However, its precision, F1 score, and recall started to decrease, with a precision of 55.77% in the first round and gradually improved to 91.65% by the 10th round. FedProx also starts with high accuracy (99.83%) and maintains it, but its other metrics, particularly precision and recall, start lower and improve over the rounds, stabilizing around 91.51% and 91.65%, respectively. In contrast, FedOpt initially had the weakest performance with an accuracy of only 0.18% in the first round, although it quickly improved to 99.93% by the third round, with similar trends in precision, F1 score, and recall.

**Figure 8:**
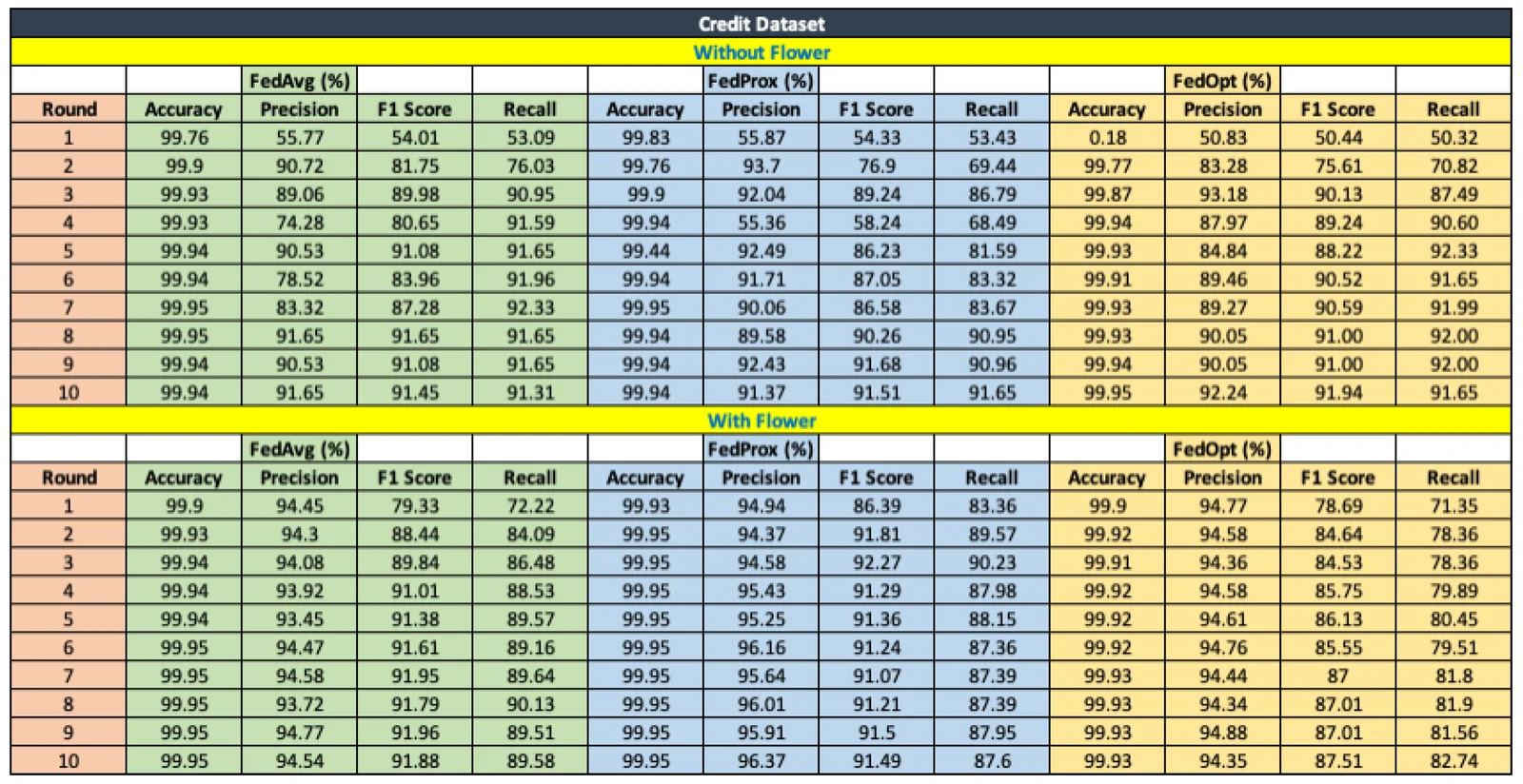
Results with and without flower framework using credit card dataset for 10 rounds.

With the flower framework, all three algorithms showed significant improvements across all metrics. FedAvg maintained its high accuracy (99.93–99.95%) while showing substantial gains in precision, F1 score, and recall, with precision starting at 79.33% and reaching 94.54% by the 10th round. FedProx also demonstrated enhanced performance, with precision, F1 score, and recall starting at higher values compared to those without the framework and stabilizing at approximately 96.37% for precision and 91.89% for recall. FedOpt’s performance is notably more stable and robust with the flower framework, achieving 99.93% accuracy consistently and improved precision, F1 score, and recall, which stabilized at higher values than without the framework. These results shown in Figure 8 clearly indicate that the flower framework enhances the stability and overall performance of federated learning algorithms when applied to the credit dataset. The consistently high accuracy and significant improvements in precision, F1 score, and recall with the flower framework underscore its effectiveness in optimizing federated learning processes for intrusion and fraud detection tasks.

### 6.3. Results Without Framework and UNSW-NB15 Dataset for Fifty Rounds

**Figure 9:**
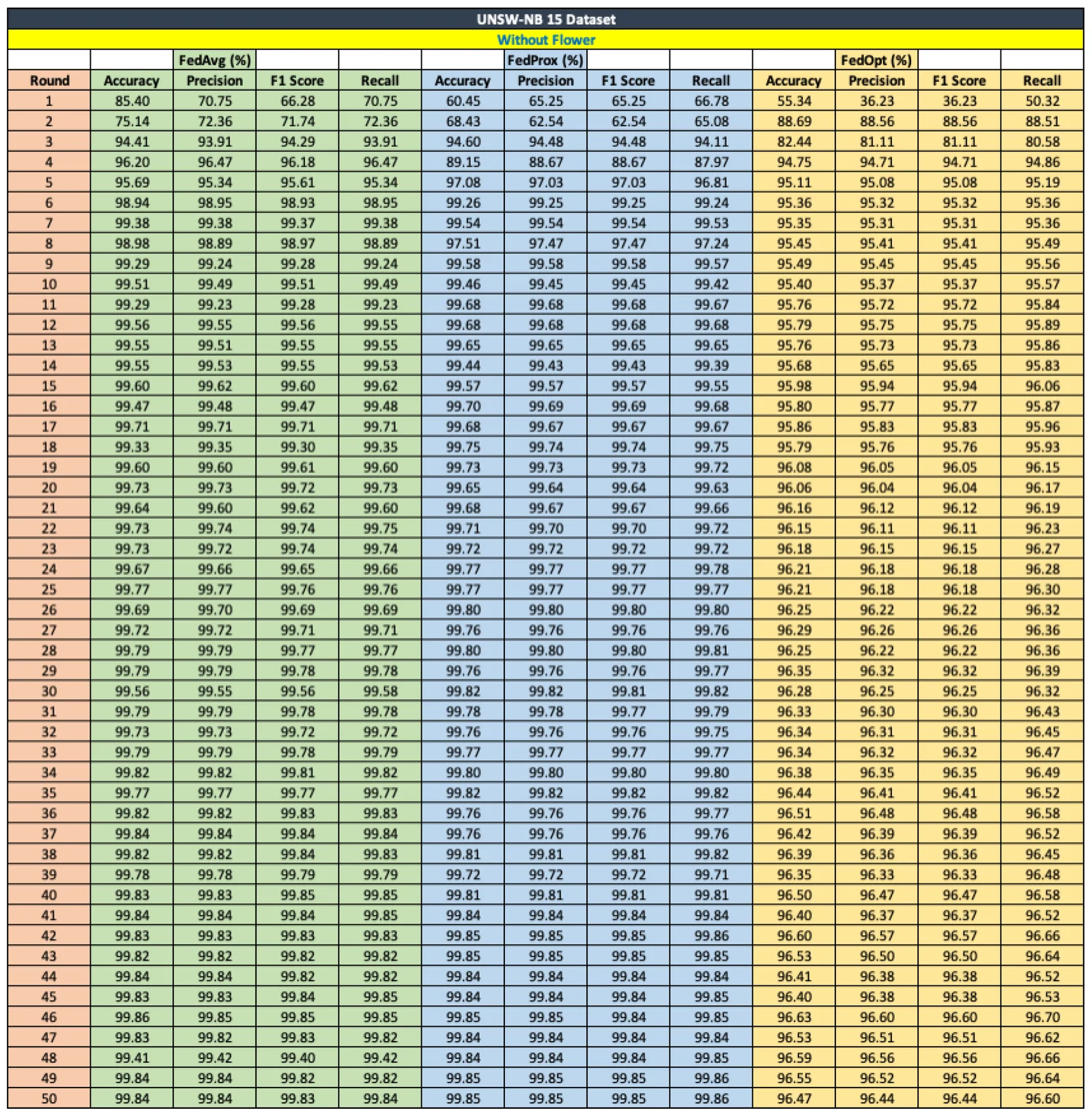
Results without flower framework using 50 rounds.

Based on the UNSW-NB15 dataset without the flower framework, Figure 9 presents the performance of the FedAvg, FedProx, and FedOpt algorithms across 50 rounds with 10 customers. The results show that all algorithms exhibited significant performance gains when used with the flower framework. The Fed AVG algorithm was found to be highly efficient. Its accuracy continued to increase as the number of rounds increased and converged at 99.84% accuracy. FedProx has undergone impressive development. Its initial accuracy was 60.45% and reached 99.85% by the 50th round. It also shows considerable improvement in memory, F1 score, and accuracy measures; recall stabilizes at 99.85%, and precision reaches 99.85%.

We compared 50 rounds of the UNSW NB-15 dataset with 10 clients using FedAvg, FedProx, and FedOpt to measure accuracy, precision, recall, area under the curve, and loss without the use of the flower framework. The FedOpt model exhibited the greatest improvement while having the lowest baseline performance. By the fifty-fifth round, the accuracy rapidly improved from 55.34% to 99.85%. FedOpt also demonstrated notable improvements in accuracy, F1 score, and memory; the precision increased from 36.23% to 96.43%, and recall reached 96.60%. The algorithms observed with the dataset revealed a significant performance improvement. The algorithms consistently performed across all the rounds used in the empirical study. Concerning intrusion detection, the algorithms demonstrated their capability to detect intrusions in a more efficient manner when associated with the flower framework.

### 6.4. Results with Framework and UNSW-NB15 Dataset for Fifty Rounds

**Figure 10:**
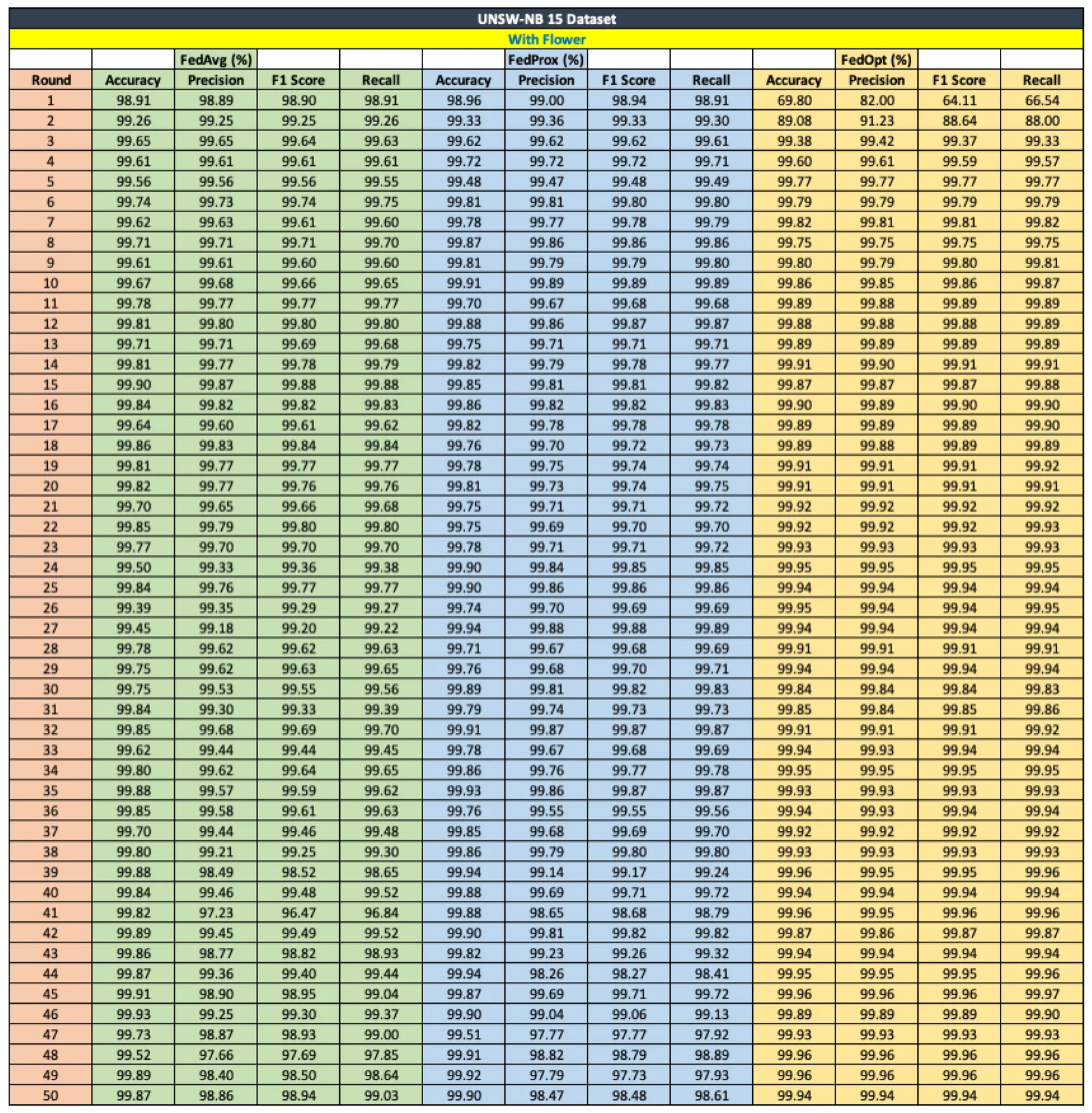
Results with flower framework using 50 rounds.

The Figure 9 and Figure 10 summarizes the performance outcomes of the three federated learning algorithms— FedAvg, FedProx, and FedOpt—applied to the UNSW-NB15 dataset over 50 rounds. The effectiveness of each algorithm was measured using four key metrics: accuracy, precision, F1 score, and recall. The results revealed distinct performance trends for each algorithm, highlighting variations in their convergence rates and overall efficiency throughout the rounds.

For FedAvg, the initial accuracy was 98.91% in the first round, which steadily increased to 99.87% after 50 rounds. This consistent improvement indicates a strong convergence over time. Similarly, the precision, F1 score, and recall start above 98.80% and climb to nearly 99.85% by the final round. The uniform enhancement across all metrics suggests that FedAvg effectively balances false positives and false negatives, maintaining a reliable performance throughout the simulation. FedProx demonstrated slightly better initial performance than FedAvg, beginning with an accuracy of 99.30% and reaching 99.90% by the end of the 50 rounds. This algorithm outperformed FedAvg in terms of early accuracy and convergence speed. The other metrics—precision, F1 score, and recall, remained high throughout the simulation. Precision starts at 99.06% and increases to 99.84%, while recall and F1 score follow similar trajectories, approaching 99.85% by round 50. FedProx’s stability from the early rounds positions it as a more consistent performer.

In contrast, FedOpt starts with a considerably lower accuracy of 69.80% but shows rapid improvement in subsequent rounds, eventually achieving an accuracy comparable to that of the other two algorithms at 99.90%. Precision, F1 score, and recall also had lower starting values—precision at 82.80%, and recall and F1 score at 64.11% and 66.54%, respectively. Despite the slower start, FedOpt’s performance aligns with those of FedAvg and FedProx by the 50th round, indicating strong convergence in the later stages. Overall, the results indicate that all three algorithms achieve high performance levels by the end of 50 rounds. FedProx stands out as the most stable from the beginning, whereas FedAvg shows steady improvement across rounds. Although FedOpt has a slower initial performance, it achieves results comparable to those of the other algorithms after more iterations, suggesting that additional rounds may be required to fully adapt to the task. In conclusion, although all algorithms perform similarly in later rounds, FedProx offers a slight advantage in convergence speed and stability, making it a favorable choice in scenarios where early performance is critical.

**Figure 11:**
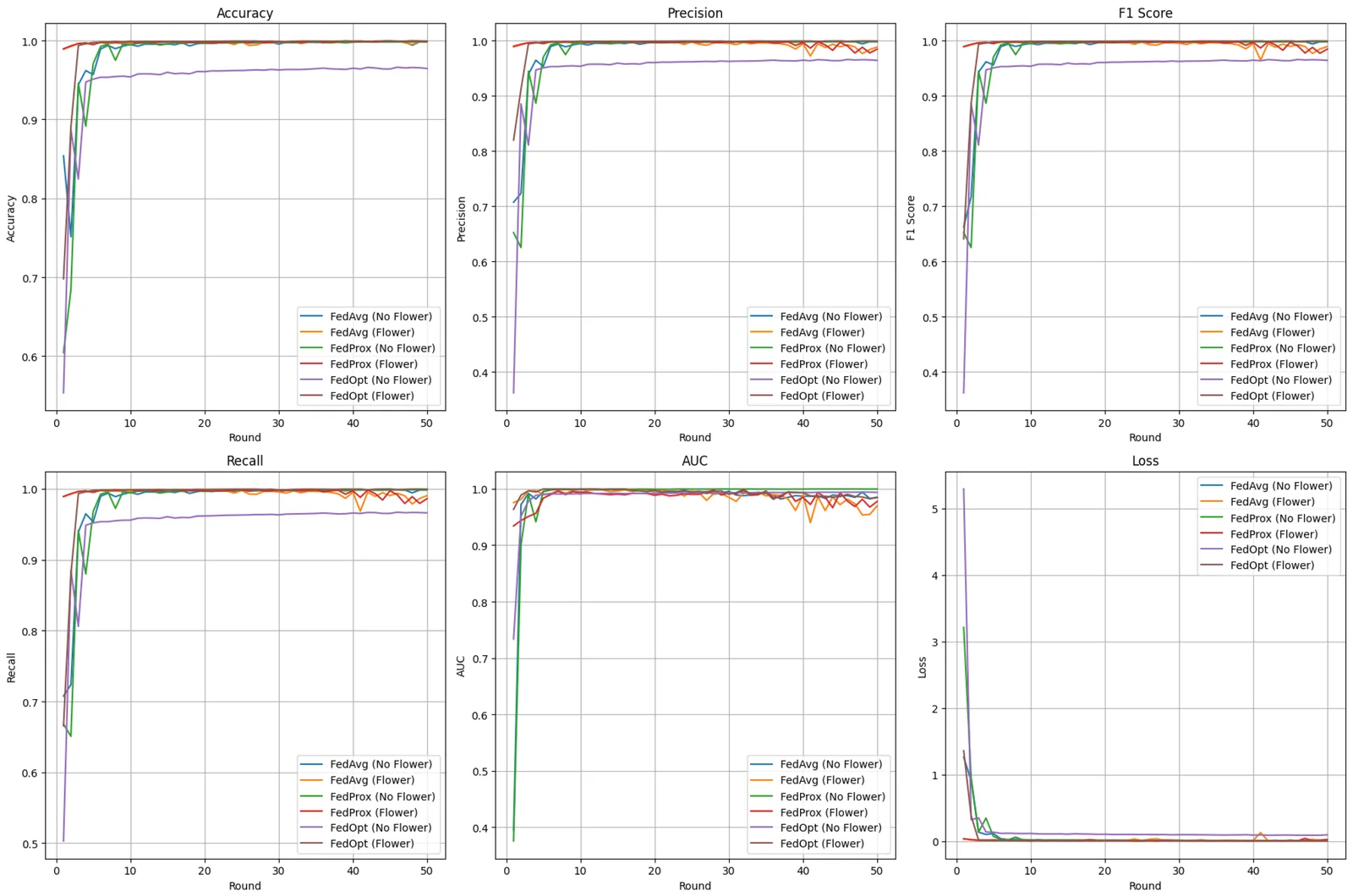
Performance comparison with and without flower framework using UNSW-NB15 dataset for 50 rounds.

The performances of various FL algorithms with and without the flower framework are shown in Figure 11. Observations were made when the framework was employed with an intrusion-detection dataset. These observations revealed that the utility of the flower framework was effectively demonstrated. When the performance is compared among all the algorithms, the algorithms can perform better when they are associated with the framework than when executed without the use of the framework.

### 6.5. Results Without Framework and Credit Card Dataset for Fifty Rounds

**Figure 12:**
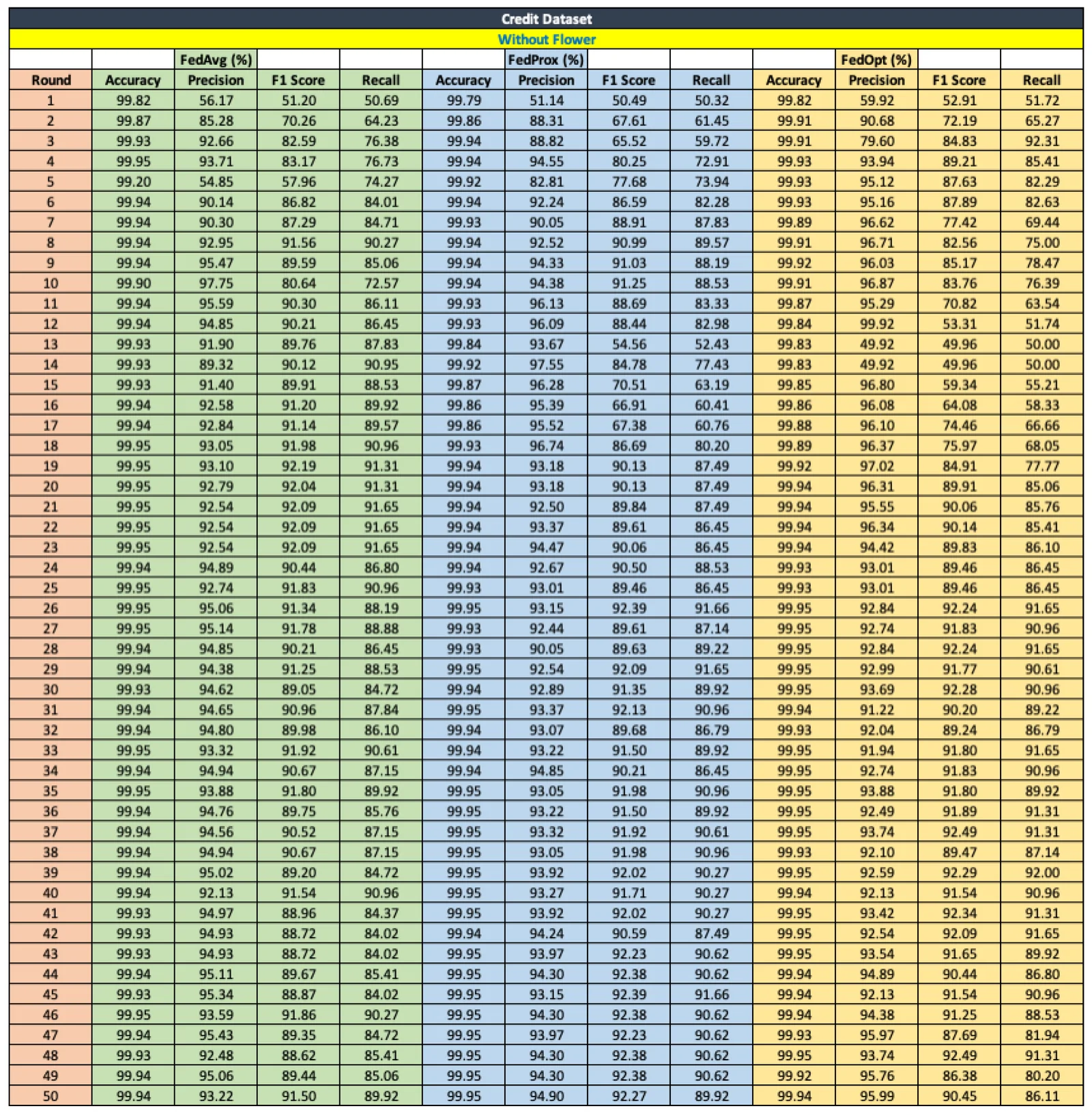
Results without flower framework using credit card dataset for 50 rounds.

The results presented in the Figure 12 highlight the performance of three federated learning algorithms—FedAvg, FedProx, and FedOpt—over 50 rounds using the credit dataset, evaluated on four key metrics: accuracy, precision, F1 score, and recall. Each algorithm demonstrated varying convergence rates and performance trends over rounds.

Starting with FedAvg, the algorithm showed strong performance across all metrics. It achieved an accuracy of 99.82% in the first round, which steadily increased to 99.94% by the 5th round and remained stable thereafter. Initially, the other metrics, such as precision (56.17%), F1 score (51.20%), and recall (50.69%), were relatively lowin the first round. However, they showed rapid improvement, particularly in the 10th round, where all three metrics surpassed 80%. By the 20th round, FedAvg stabilized with all metrics above 90%, demonstrating its reliability and consistent improvement over time. FedProx exhibited a slightly faster improvement rate compared to FedAvg, with an accuracy of 99.79% in the first round and quickly stabilized at 99.94%. Precision, F1 score, and recall showed similar growth patterns. While the initial values for precision (51.14%), F1 score (50.49%), and recall (50.32%) were comparable to those of FedAvg, FedProx quickly surpasses the 90% mark for these metrics by the 10th round. Throughout the remainder of the rounds, FedProx maintained a highly stable performance, consistently scoring above 90% for all key metrics, indicating that it offers a strong balance of precision, recall, and overall accuracy throughout the simulation.

In contrast, FedOpt started with significantly lower values for precision (59.92%), F1 score (52.91%), and recall (51.72%) than FedAvg and FedProx. Despite these low initial values, FedOpt showed continuous improvement as the rounds progressed. In the 10th round, its precision reached 83.76%, while the F1 score and recall also showed significant gains, increasing to 76.13% and 73.64%, respectively. FedOpt stabilizes around the 90% range for all key metrics by the 20th round but generally lags slightly behind the other two algorithms in terms of early-round stability and performance consistency. However, its ability to close the gap in later rounds suggests that FedOpt may be more suited to tasks requiring extended rounds for optimal convergence. Overall, the results indicate that all three algorithms converged to high performance levels by the 50th round, with accuracy consistently exceeding 99.9%. However, FedProx stands out as the most stable and balanced performer, showing faster and more consistent improvement across all metrics from the early rounds. While also performing well, FedAvg showed a slightly more gradual improvement. FedOpt, although slower to converge in the early rounds, eventually achieved comparable performance to the other two algorithms. In summary, although all three algorithms perform effectively in the final rounds, FedProx offers an advantage in terms of convergence speed and stability, making it a favorable option in scenarios where early performance is critical.

### 6.6. Results with Framework and Credit Card Dataset for Fifty Rounds

**Figure 13:**
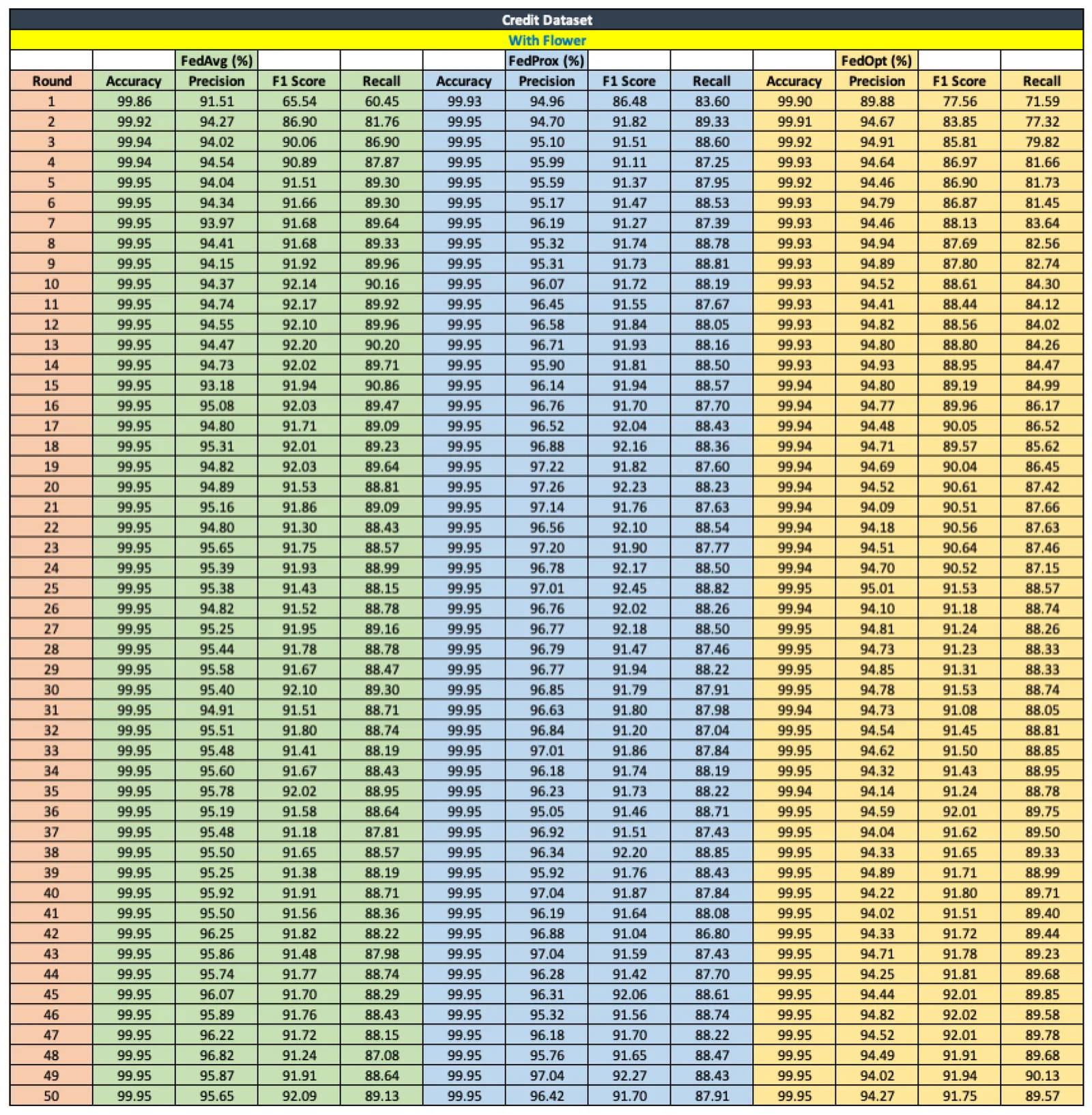
Results with flower framework using credit card dataset for 50 rounds.

Figure 13 summarizes the outcomes of applying three federated learning algorithms—FedAvg, FedProx, and FedOpt—to the credit dataset using the flower framework over 50 rounds. The effectiveness of each algorithm was measured using four key metrics: accuracy, precision, F1 score, and recall. The results highlight the differences in how quickly the algorithms converge and their overall performance across rounds. The FedAvg algorithm demonstrated a strong and consistent performance throughout the simulation. Its accuracy begins at 99.86% in the first round and stabilizes at 99.95% early, maintaining this level up to 50 rounds. Initially, FedAvg recorded precision, F1 score, and recall values of 91.51%, 65.54%, and 60.45%, respectively, indicating robust early performance, particularly in precision. By the 10th round, these metrics improved significantly, with both F1 score and recall exceeding 90%. From round 20 onward, all metrics remained high and stable, indicating strong convergence.

FedProx exhibited an excellent performance during the simulation. Its accuracy started at 99.93% and quickly reached 99.95% in the early rounds. The initial precision, F1 score, and recall were 94.96%, 86.48%, and 83.60%, respectively, indicating a stronger early performance than FedAvg. These metrics remained high from the outset and continued to improve. By round 10, the F1 score and recall surpassed 90%, and this stability was maintained throughout the remaining rounds with minimal fluctuations. FedProx demonstrated one of the most consistent performances across all metrics, indicating excellent early convergence and high stability. FedOpt, while performing well in terms of accuracy, starts with slightly lower initial metrics than FedAvg and FedProx. Its accuracy begins at 99.89% and stabilizes at 99.94% by the 10th round. However, the initial precision, F1 score, and recall were lower, starting at 88.88%, 77.56%, and 71.59%, respectively. These metrics gradually improved over the rounds, with notable increases in the F1 score and recall, which exceeded 90% by round 20. Although FedOpt achieves a strong overall performance by the 50th round, it shows slower early convergence than the other two algorithms.

**Figure 14:**
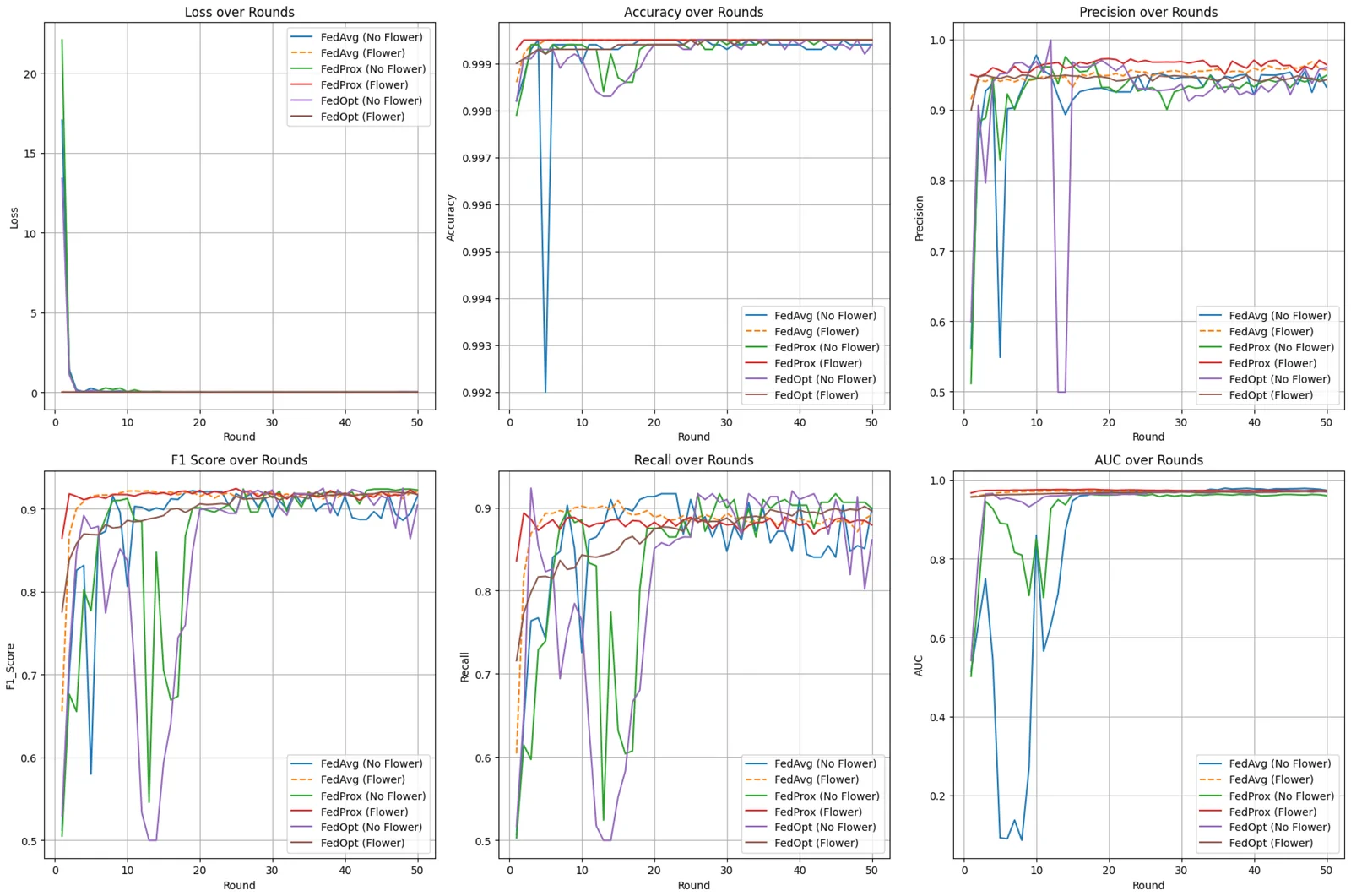
Performance comparison with and without flower framework using credit card dataset for 50 rounds.

In conclusion, all three algorithms performed exceptionally well by the end of the 50 rounds, consistently achieving accuracy levels above 99.9%. FedProx stands out as the most stable performer, offering superior early-round performance and consistency across all metrics. FedAvg showed steady improvements in the initial rounds, reaching a strong performance by the middle of the simulation. Despite a slower start, FedOpt catches up over time and attains comparable performance in later rounds. These results suggest that FedProx may be particularly suitable for scenarios in which early stability and performance are crucial, whereas FedAvg and FedOpt also provide reliable results with additional training rounds as shown in Figure 14.

The empirical study made with credit card dataset with all the algorithms considering the flower framework using 10 clients and 50 rounds could provide significant insights. Each algorithm was executed for 50 rounds, and all clients participated in the learning process. The convergence point in the last round of FedProx achieved an accuracy of 99.95%. FedOpt also showed a significant performance gain with 99.39% accuracy. The experiments using the credit card dataset observe all algorithms without using the flower framework. The empirical study and results revealed that all algorithms can achieve significant performance gains when the framework is used. The rationale behind this is that the framework provides inherent benefits in dealing with various complexities associated with collaborative learning.

### 6.7. Overall Performance Comparison with UNSW NB-15 Dataset for Fifty Rounds

The performances of all algorithms were evaluated with and without the flower framework. This section presents a performance comparison among the algorithms when 10 clients are involved in the learning process with 50 rounds. The results reveal the utility of the framework in improving the performance of the algorithms. The FedAvg algorithm performed better using the flower framework, with 98.63% accuracy. The performance of FedProx improved up to 97.91%, whereas the FedOpt exhibited 98.64% accuracy.

**Figure 15:**
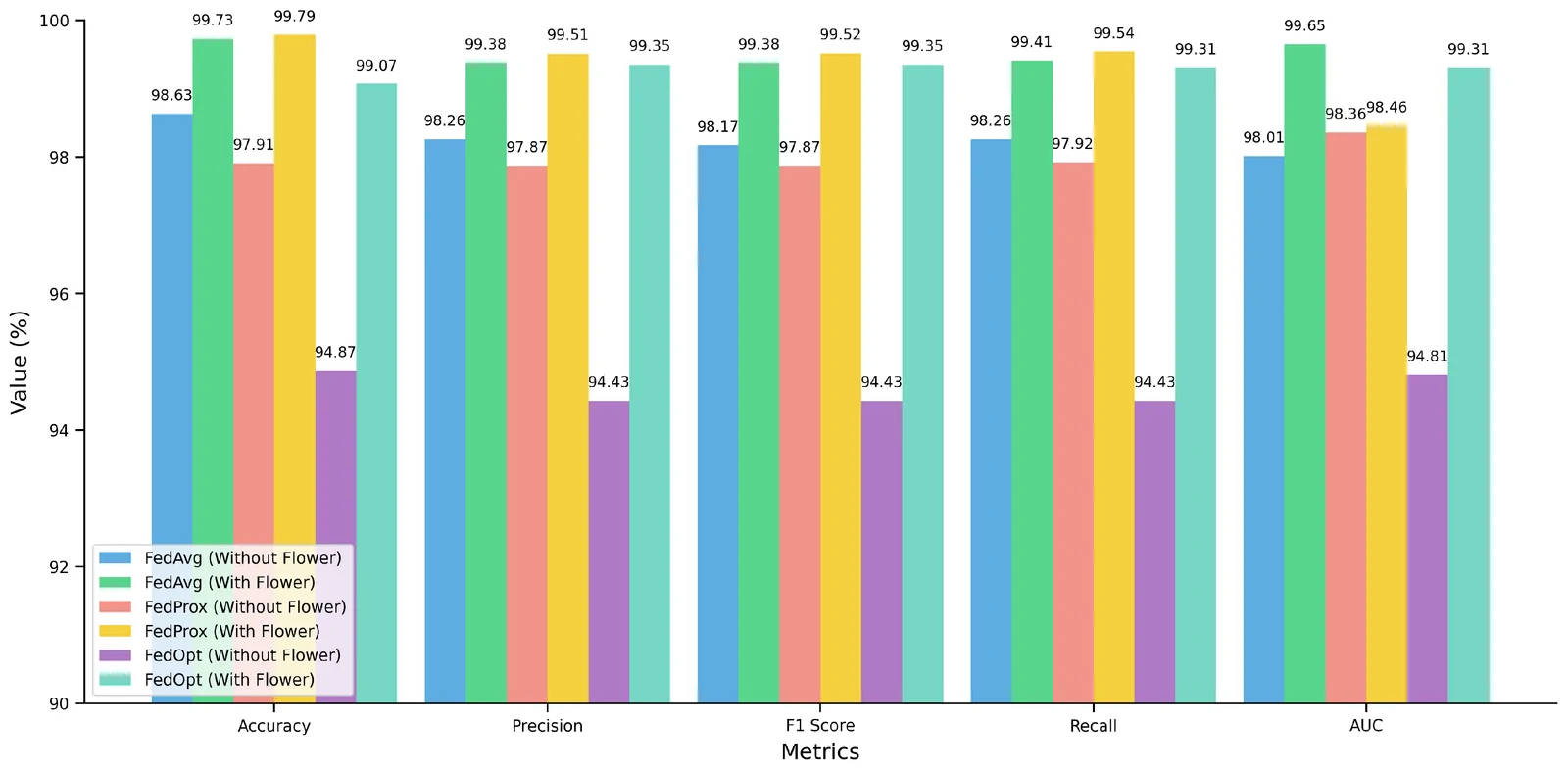
Overall performance with UNSW-NB15 dataset for 50 rounds.

The average acc, prec, f1, recall, AUC, and Loss using FedAvg, FedProx, and FedOpt for 50 rounds with 10 clients for the UNSW NB-15 dataset with and without the flower framework are provided. The introduction of the flower framework resulted in significant improvements. Using 50 rounds with the flower framework, FedAvg further improved the accuracy to 99.73%, precision to 99.38%, F1 score to 99.38%, recall to 99.41%, and an AUC to 98.65%. FedProx maintained the best performance with an accuracy of 99.79%, precision of 99.51%, F1 score of 99.52%, recall of 99.54%, and AUC of 98.46%. FedOpt achieved remarkable metrics with an accuracy of 99.07%, precision of 99.35%, F1 score of 98.95%, recall of 98.98%, and AUC of 99.31. These findings as shown in Figure 15 underscore the substantial performance enhancements enabled by the flower framework, making it a valuable tool for optimizing federated learning algorithms in intrusion detection tasks.

### 6.8. Overall Performance Comparison with Credit Card Dataset for Fifty Rounds

**Figure 16:**
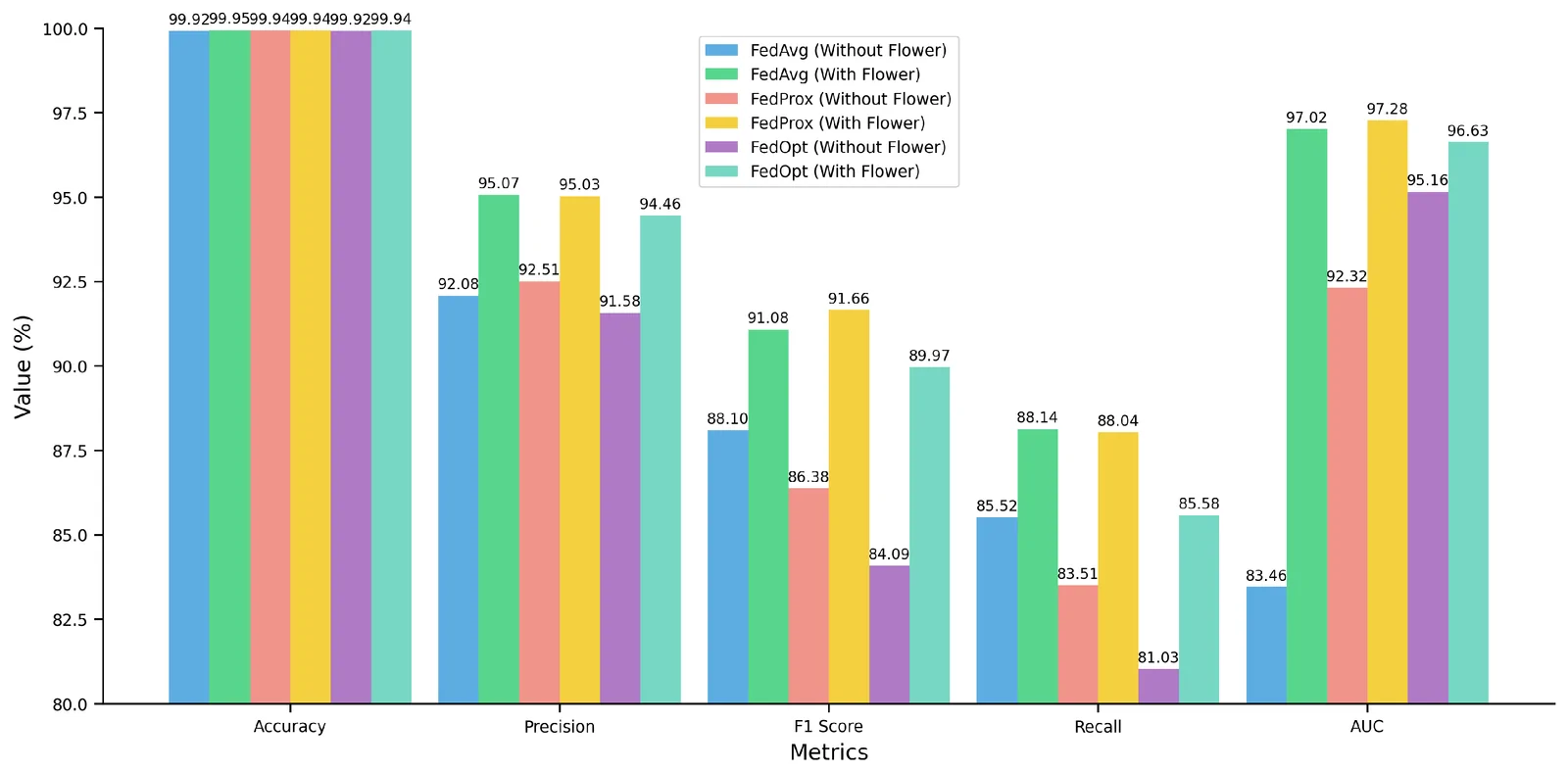
Overall performance with credit card dataset for 50 rounds.

The Figure 16 provides a comparative analysis of the average accuracy, precision, F1 score, recall, and AUC of the FedAvg, FedProx, and FedOpt algorithms over 50 rounds with 10 clients, using the credit card dataset, both with and without the flower framework. The results demonstrate clear performance enhancements when the flower framework is employed. Training with 50 rounds without the flower framework resulted in minor improvements in accuracy for FedAvg (99.92%) and FedProx (99.93%), but with mixed results for other metrics, indicating a plateau effect.

Comparing average acc, perc, f1, recall, AUC, and Loss using FedAvg, FedProx, and FedOpt for 50 rounds with 10 clients for the credit card dataset with and without the flower framework was done. The introduction of the flower framework resulted in substantial improvements across all metrics. FedAvg and FedProx attained a nearly flawless accuracy of 99.95% after 50 rounds utilizing the flower framework. FedAvg demonstrated 95.07% precision, 91.08% F1 score, 88.14% recall, and 97.02 AUC. FedProx performed better, with 96.34% accuracy, 91.66% F1 score, 88.04% recall, and 97.28% AUC. With an F1 score of 89.97%, recall of 86.58%, accuracy of 99.94%, precision of 94.46%, and AUC of 96.63%, FedOpt also demonstrated notable gains. These observations reveal that the flower framework made a significant contribution in leveraging the performance of the algorithms used for collaborative learning. With respect to both case studies, such as fraud detection in credit card transactions and also intrusion detection, significant performance improvement were achieved with the flower framework.

## 7. Conclusions and Future Scope

The methodology proposed in this study exploits the flower framework to leverage the performance of FL algorithms. The empirical study involved two case studies with benchmark datasets covering credit card fraud and intrusion detection. The algorithms proposed and used along with the flower framework were evaluated with an experimental setup where 10 clients were used, and 50 rounds are considered for the study. Observations were made to determine the performance of the algorithms with and without the flower framework. The algorithms were executed for two case studies without using the framework. Even without using this framework, the algorithms can provide considerable performance in detecting credit card fraud and inclusions efficiently. However, when the algorithms are executed with the flower framework, their performance is further improved because the framework is capable of helping the algorithms with various optimizations. This study highlights the significance of using cutting-edge frameworks, such as flower, to further improve the performance of federated learning algorithms and consequently make a substantial contribution to the evolution of security and privacy in digital infrastructures. Federated learning has many opportunities for storage in the future. The general FL framework and approach proposed in this study can be incorporated to enable collaborative learning in sensitive domains such as biomedicine, healthcare, and finance. Data are susceptible in the healthcare domain, and further study is required to realize a privacy-preserving system for collaborative machine learning. In finance, it is essential to maintain objectivity in the analysis because the data involve personal and sensitive information.

In the future, FL can be further investigated in the context of IoT workflow applications that benefit from energy and computing. The possibility of exploiting FL in autonomous vehicle research and IoT use cases, such as in smart homes and smart cities, is also possible. Federated learning can also be further investigated to ensure personalization and auto-scaling in terms of developing recommender systems and the efficient usage of smart handheld devices. In the future, it is also possible to use federated learning toward cross-industry collaboration to have a synergic approach to obtaining the required business intelligence. There is also scope for future research in which model efficiency can be enhanced, communication costs can be reduced, and handling of heterogeneity and personalization of models across the sites can be addressed. In the future, it will also be possible to develop interoperability standards for federated learning, considering required ethics and governance frameworks.

## Figures and Tables

**Table 1:** Comparison of related federated learning studies.

Reference	FL Architecture	Algorithms	Datasets	Key Contributions
Sun et al. [10]	Hierarchical FL	Transformer-based IDS	5G Smart Grid Data	Improved IDS performance in smart grids using transformer models and hierarchical FL
Hamdi [11]	Standard FL	FedAvg, FedProx, etc.	CSE-CIC-IDS2018	Per-client evaluation to understand privacy-performance trade-offs in FL for IoT
Begum et al. [12]	Blockchain-driven FL	FedAvg	IoMT Network Data	Enhanced security in FL through blockchain integration for intrusion detection
Yang et al. [13]	Horizontal FL	Custom FL methods	Credit Card Transactions	Addressed data insufficiency in credit card fraud detection using FL
Kumar et al. [14]	Edge intelligence FL	Artificial Immune System-based IDS	KDD-99, CIFAR-10	Privacy-preserving IDS in edge computing for 5G networks
Dasari and Kaluri [19]	Standard FL using flower	Not specified	Financial Data	Privacy-preserving approach in financial sectors using flower framework
Chen et al. [20]	Asynchronous FL	Custom Deep Learning Models	MNIST, HAR	Communication-efficient FL with asynchronous updates using flower framework
Our Work	Standard FL using flower	FedAvg, FedProx, FedOpt	UNSW-NB15, Credit Card Data	Empirical evaluation of FL algorithms using flower for intrusion and fraud detection across diverse datasets

**Table 2:** Summary of key features of papers utilizing the flower framework for federated learning.

Reference	Datasets	Flower	Rounds	Architecture	FL Settings
[50]	-	Yes	-	None	Client impact
[51]	MNIST	Yes	-	None	Device eval
[52]	COVID-19	Yes	-	CNN	Client variation
[53]	MIMIC	Yes	-	MLP	Hospital study
[54]	Chest X-ray	Yes	-	ANN	Cloud training
Our Work	UNSW-NB15	Yes	50	CNN-IIDNet	IDS, Fraud

**Table 3:** Summary of federated deep learning methods.

Reference	Method/s	Dataset/s	Performance Metrics	Outcome/s
[19]	CNN, LSTM, CNN-LSTM	IoTID20	Accuracy, precision, sensitivity, specificity, F1-score	Proposes IoT IDS combining CNN, LSTM, and PSO, showing robust intrusion detection and effective attack classification.
[20]	CNN-GRU	Real-world gas pipelining system dataset	Accuracy, precision, recall, F1-score	Proposes DeepFed for cyber threat detection in industrial CPSs, outperforming state-of -the-art models in all performance metrics
[21]	GRU, RF (as an ensembler)	Modbus-based network dataset	Accuracy, training time	Proposes IoT IDS using federated learning, achieving competitive intrusion detection with CNN and RNN models compared to centralized systems.
[27]	AE	Gas pipeline SCADA system dataset	F1-score, accuracy	Federated anomaly detection with GRUs for IoT networks shows higher accuracy in attack classification than non- federated approaches.
[]	CNN, RNN	NSL-KDD	Accuracy, Precision, detection rate, F1-score, communication cost	Discusses general advantages of federated learning for intrusion detection without specific study outcomes.
[55]	Cloud based NN	CIC-IDS 2017 and CIC-IDS 2018	Accuracy, F1-score, True-Positive Rate (TPR), False-Positive Rate (FPR)	Presents lightweight IDS for IIoT using ensemble classifiers, leveraging instance- based transfer learning and FL for privacy- preserving knowledge sharing.
[29]	FedKD utilizes knowledge distillation to minimize communication overhead	CIFAR-10 and NF-BoT-IoT-v2	Communication overhead, model accuracy, convergence rate	Significant reduction in communication cost while maintaining model accuracy.
[31]	Compresses local data using feature extraction before aggregation	Wine Quality Dataset, Rice MSC Dataset	Model accuracy, data compression ratio, communication overhead	Efficient communication and improved model.
[37]	CNN, 2NN	MNIST	Accuracy, number of communication rounds	FL has significant promise, as high-quality models can be trained using relatively few rounds of communication.
[45]	AE, DBN	NSL-KDD	Accuracy, precision, recall	Proposes IoT IDS using FL, achieving competitive intrusion detection with CNN and RNN models compared to centralized systems.

**Table 4:** Analysis of federated learning frameworks.

Framework	Communication Overhead	Bandwidth Usage	Round Efficiency	Latency	Data Leakage	Security Against Attacks	Client Anonymity
FATE	Moderate	Moderate	High	Low	Low	High	Moderate
Flower	Low	Low	High	Low	Low	High	High
PySyft	High	High	Moderate	Moderate	Low	High	High
Open Federated Learning	Moderate	Moderate	Moderate	Moderate	Moderate	High	Moderate
Tensor Federated Learning	Moderate	Moderate	High	Low	Low	High	Moderate
Substra	Moderate	Moderate	High	Low	Low	High	High
Fed ML	High	High	Moderate	Moderate	Low	Moderate	Moderate
LEAF	Low	Low	High	Low	Low	High	High
NVIDIA FLARE	Moderate	Moderate	High	Low	Low	High	Moderate
IBM Federated Learning	Moderate	Moderate	High	Low	Low	High	High

**Table 5:** Details of enhanced CNN model.

Layer	Type	Details
Input Layer	-	9 × 9 matrix with 77 features and 4 zero pads
Convolutional Layer 1	Conv2D	9 × 9 kernel, 16 feature maps
Max Pooling Layer 1	MaxPooling2D	Reduces spatial dimensions
Convolutional Layer 2	Conv2D	5 × 5 kernel, 32 feature maps
Max Pooling Layer 2	MaxPooling2D	Reduces spatial dimensions
Convolutional Layer 3	Conv2D	3 × 3 kernel, 32 feature maps
Flatten Layer	Flatten	Transforms 2D feature maps into a single vector
Fully Connected Layer 1	Dense	Pattern learning and classification
Output Layer	Dense (Softmax)	Neurons for each class: Benign, DDoS, DoS, Portscan, and Webattack, representing probabilities

## Data Availability

The dataset UNSW-NB15 used for this research is available at https://research.unsw.edu.au/projects/unsw-nb15-dataset (accessed on 1 June 2023).

## Supplementary Materials

The Code Availability, Repository Organization, Experimental Environment, and Reproducibility Statement can be downloaded at: https://www.mdpi.com/article/10.3390/s25103043/s1.
