# Correction: Deshmukh et al. Enhancing Privacy in IoT-Enabled Digital Infrastructure: Evaluating Federated Learning for Intrusion and Fraud Detection. *Sensors* 2025, *25*, 3043

**DOI:** 10.3390/s26144542

**Published:** 2026-07-17

**Authors:** Amogh Deshmukh, Peplluis Esteva de la Rosa, Raul Villamarin Rodriguez, Sandeep Dasari

**Affiliations:** 1 School of Technology, Woxsen University, Hyderabad 502345, India; peplluis.esteva@woxsen.edu.in (P.E.d.l.R.); dasari.sandeep@woxsen.edu.in (S.D.); 2 School of Business, Woxsen University, Hyderabad 502345, India; raul.rodriguez@woxsen.edu.in

**Text Correction**

There were some typos and misunderstandings of some expressions in the original publication [1].

1. A correction has been made to the Abstract:

**Abstract:** Challenges in implementing machine learning (ML) include expanding data resources within the finance sector. Banking data with significant financial implications are highly confidential. Diverse breaches and privacy violations can result from a combination of user information from different institutions for banking purposes. To address these issues, federated learning (FL) using a flower framework is utilized to protect the privacy of individual organizations while still collaborating through separate models to create a unified global model. However, joint training on datasets with diverse distributions can lead to suboptimal learning and additional privacy concerns. To mitigate this, established FL algorithms such as federated averaging (FedAvg), federated proximal (FedProx), and federated optimization (FedOpt), previously introduced in the literature, are evaluated in our study. These methods work with data locality during training at local clients without exposing data, while maintaining global convergence to enhance the privacy of local models within the framework. In this analysis, the UNSW-NB15 and credit datasets were employed, utilizing precision, recall, accuracy, F1-score, ROC, and AUC as performance indicators to demonstrate the effectiveness of applying FedAvg, FedProx, and FedOpt within the flower framework. The algorithms considered were subjected to an empirical study, which revealed significant performance benefits when using the flower framework. Consequently experiments were conducted over 50 rounds using the UNSW-NB15 dataset, which achieved accuracies of 99.87% for both FedAvg and FedProx and 99.94% for FedOpt. Similarly, with the credit dataset under the same conditions, FedAvg and FedProx achieved accuracies of 99.95% and 99.94%, respectively, while FedOpt also achieved comparable accuracy. These simulation-based results suggest that the proposed framework is effective on widely used benchmark datasets and, with further validation in deployed environments, has the potential to support secure and privacy-preserving collaborative machine learning across various domains.

2. In the 1st paragraph of Section 1. The content “FL is advantageous in environments with heterogeneous data, devices, settings, and communication protocols” has been updated to “FL is advantageous in environments with heterogeneous data, devices, settings, and communication protocols, a property that has been highlighted in the foundational FL literature [1]”.

3. In the 4th paragraph of Section 1. The corrected paragraph appears below:

This study focuses on utilizing the flower framework to evaluate various FL algorithms in solving problems such as intrusion detection [2] and fraud detection [3] in credit cards. The key contributions of this study are as follows:

1. Methodology Proposal: Introducing a methodology based on the flower framework for empirical studies of existing FL algorithms using diverse datasets.
2. Algorithm Integration: Integrating well-established FL algorithms (FedAvg, FedProx, and FedOpt, as originally introduced in the FL literature) into the flower framework together with our enhanced CNN model (IIDNet), and configuring them for the non-IID data encountered in intrusion and fraud detection. We do not claim novelty of the underlying algorithms themselves; the novelty lies in their joint implementation and empirical study within this framework.
3. Empirical Evaluation: Conducting an empirical study with a prototype application, executing different algorithms within the framework, and evaluating their performance.

4. In Section 5.2. The corrected 5th and 6th paragraphs appear below:

A convolutional neural network (CNN) was used to classify network traffic and credit card transactions as shown in Figure 2. The CNN architecture included input layers to receive preprocessed data, convolutional layers (Conv1D/Conv2D) with filters (e.g., 32, 64) and kernel sizes (e.g., 3 × 3, 5 × 5) to capture spatial features, and ReLU activation functions to introduce non-linearity. MaxPooling layers were used to reduce the spatial dimensions and computational complexity. Dropout was also applied during training (with a dropout rate of 0.25 after the pooling stages and 0.5 before the output layer) as a standard regularization technique to mitigate overfitting; these regularization layers are omitted from the simplified architecture diagram in Figure 2 and the layer summary in Table 5 purely for readability, but they were part of the model used during training. Dense layers (e.g., 128 and, 64 units) performed classification based on features extracted by convolutional layers, with ReLU activation functions in hidden dense layers and softmax or sigmoid activation functions in the output layer for multi-class or binary classification, respectively. It is worth noting that the input representation is a 9 × 9 matrix containing 77 feature values (with four zero-pads to complete the grid). The UNSW-NB15 dataset originally provides 42 features, and the expansion to 77 occurs because categorical attributes (such as proto, service, and state) are one-hot encoded during preprocessing, which is a standard feature engineering step that converts each categorical attribute into several binary columns.

The hyperparameters and training setup included a batch size of 32, 50 global communication rounds (with a small number of local epochs per round at each client), a learning rate of 0.001, and the use of the Adam optimizer both on the client side and, where applicable (for example, in the server-side update step of FedOpt), on the server side. The loss functions used were binary cross-entropy for binary classification tasks (the credit card fraud detection case, where the label indicates fraudulent or legitimate transactions) and categorical cross-entropy for multi-class classification tasks (the UNSW-NB15 case, where the label denotes the attack category as reflected in Table 5). Early stopping with a patience of five epochs was employed to prevent overfitting by monitoring the validation loss, and model checkpointing was used to save the best model based on the validation loss. For FedProx, the proximal regularization coefficient *μ* was set to a small constant value (0.01), consistent with values commonly reported in the literature for similarly sized models and datasets. For our federated setup, the training data were partitioned horizontally across ten clients using a non-IID split, so that each client held a subset of the samples while all clients shared the same feature space; in every round, all ten clients participated (i.e., *K* = 10) rather than a randomly sampled subset, in order to keep the experimental comparison across algorithms and rounds stable. Table 5 presents the layers involved in the enhanced CNN model. Further details on code availability, repository organization, the experimental environment, and reproducibility are provided in the Supplementary Materials.

5. In 1st paragraph of Section 6. The contents “It is worth clarifying that the numerical results reported here are not directly comparable with those of our prior centralized work [56] that used the same enhanced CNN backbone, as the training paradigm (federated vs. centralized), data partitioning, and optimization dynamics differ; any improvements observed here should therefore be interpreted in the context of the federated setting rather than as a head-to-head improvement over the earlier centralized model.” have been added after the 2nd sentence.

6. A “Supplementary Materials” section is added, and it appears below:

**Supplementary Materials:** The Code Availability, Repository Organization, Experimental Environment, and Reproducibility Statement can be downloaded at: https://www.mdpi.com/article/10.3390/s25103043/s1.

**References Correction**

The original Reference 54 has been updated as Reference 1. With this correction, the order of some references has been adjusted accordingly. 

The authors state that the scientific conclusions are unaffected. This correction was approved by the Academic Editor. The original publication has also been updated.
