# Supplementary material for: Enhancing Privacy in IoT-Enabled Digital Infrastructure: Evaluating Federated Learning for Intrusion and Fraud Detection
Source: Sensors (Basel). 2025 May 12;25(10):3043. doi: 10.3390/s25103043 (PMC12114921; doi:10.3390/s25103043)
Supplement: Supplementary file 1 [file sensors-25-03043-s001.zip › sensors-3536071-Supplementary Materials.pdf]

Supplementary Materials

S1. Code Availability

The complete source code used to conduct all experiments reported in this paper is publicly available in a GitHub repository:

GitHub Repository:
https://github.com/amoghpesit/privacy-preserving-federated-learning-ids

The repository includes implementations of federated learning algorithms for intrusion detection and fraud detection tasks.

S2. Repository Organization

The repository is organized as follows:

- **CreditCard/No_Flower/**
  Standalone Python implementations of FedAvg, FedProx, and FedOpt algorithms applied to the Credit Card Fraud dataset without using federated learning frameworks.
- **CreditCard/Flower/**
  Flower-based implementations of FedAvg, FedProx, and FedOpt for the Credit Card Fraud dataset.
- **UNSW/No_Flower/**
  Standalone Python implementations of FedAvg, FedProx, and FedOpt algorithms applied to the UNSW-NB15 intrusion detection dataset without using federated learning frameworks.
- **UNSW_NB15/**
  Flower-based CNN implementations of FedAvg, FedProx, and FedOpt evaluated on the UNSW-NB15 intrusion detection dataset for 10 and 50 communication rounds.
- **datasets/**
  Dataset access information and links.

S3. Experimental Environment

All experiments were conducted using Python and TensorFlow. Federated learning simulations were implemented using the Flower framework where applicable. Standard machine learning libraries such as NumPy, Pandas, and scikit-learn were used for data processing and evaluation.

S4. Reproducibility Statement

Due to stochastic optimization, random initialization, and client sampling, numerical results may vary slightly across executions. However, overall trends and comparative performance between algorithms remain consistent with the findings reported in the manuscript.
